# The influence of dual-energy computed tomography image noise in proton therapy treatment planning

**DOI:** 10.1016/j.phro.2023.100493

**Published:** 2023-09-20

**Authors:** Torbjörn Näsmark, Jonas Andersson

**Affiliations:** Department of Radiation Sciences, Radiation Physics, Umeå University, SE-901 85 UMEÅ, Sweden

**Keywords:** Proton therapy, Stopping power mapping, Dual-energy computed tomography

## Abstract

**Background and purpose:**

In proton therapy, a 3.5% margin is often used to account for proton range uncertainties, of which computed tomography (CT) image noise is assumed to contribute 0.5%. This work evaluates the noise-sensitivity of three dual-energy computed tomography (DECT)-based methods for mapping proton stopping power relative to water (SPR): Näsmark & Andersson (N&A), Landry-Saito (L-S), and the commercial application DirectSPR.

**Methods and materials:**

DECT image data of a CIRS-062M phantom was acquired with CT scanners from two different vendors. Acquisitions were repeated 30 times to account for intra- and inter-scan variations. SPR maps were generated with the three DECT-based methods and range simulated in a commercial treatment planning system.

**Results:**

Noise in input data was amplified in L-S SPR maps, kept level with DirectSPR, while N&A compressed noise overall but displayed sensitivity to the choice of input data, potentially leading to increased noise levels. In our simulations, only N&A improved upon the assumed 0.5% noise contribution to range uncertainty on one scanner. On the other scanner, uncertainties exceeded 0.5% for all three methods. Mitigation of this issue was demonstrated by using a method employing virtual mono-energetic images as input. Increasing imaging radiation dose, as expected, alleviates the problem, while applying noise reduction only helped to a lesser extent.

**Conclusions:**

While range uncertainty due to noise is small compared to other contributions, it becomes more important as we move towards smaller treatment margins and the noise-sensitivity of SPR mapping methods should be carefully estimated and considered before clinical implementation.

## 1 Introduction

Accurate mapping of proton stopping power relative to water (SPR) and range estimation are essential to utilize the potential benefits of proton therapy over conventional radiotherapy [1], [2], [3]. The combined uncertainty in proton range estimation has been reported in the literature as 3.5%+2.0 mm (1.5 standard deviations), with a contribution of 0.5% from computed tomography (CT) imaging and calibration [4], [5]. Multiple dual-energy computed tomography (DECT)-based methods to improve SPR mapping have been suggested in the literature, reporting SPR residual root mean square errors (RMSE) between 0.5% and 1.5% [6], [7], [8], [9], [10], [11]. These methods use native low and high kilovoltage (kV) images as input and have been developed and evaluated on dual-source DECT scanners. Möhler *et al.* showed that many of these methods are mathematically equivalent and suggested that future work in the field should focus on improving calibration rather than developing new variations of the same formalism [12].

Without resorting to sequential scans, native low and high kV-images are not available on fast-kV switching or dual-layer detector DECT scanners. Therefore, in a previous work we introduced a general method for SPR mapping, i.e., compatible with all commercially available DECT and photon counting CT scanners, that uses DECT-generated virtual monoenergetic images (VMI) as input [13]. This method demonstrated potential improvement upon the conventional method by Schneider *et al.* [16], with SPR RMSEs of 7.2% (lung tissue), 0.4% (soft tissue), and 0.8% (bone), compared to 5.9% (lung tissue), 0.9% (soft tissue), and 5.1% (bone), for our implementation of the conventional method [13].

This work evaluates the noise-sensitivity of three different DECT-based methods for SPR mapping: (1) a general method (N&A) [13]; (2) a method based on Landry *et al*. and Saito *et al*. (L-S) [14], [15]; and (3) the application DirectSPR (Siemens Healthineers, Erlangen, Germany) [17]. N&A uses VMIs as input, while L-S and DirectSPR both use native low and high kV-images. Out of the kV-based methods described in the literature, L-S was chosen as it has been evaluated in several other works [3], [18], [19]. DirectSPR was included as it is the only commercially available application of its kind.

Due to the stochastic nature of x-rays and CT detectors, images acquired under identical circumstances may be expected to differ from each other. While intra-scan noise sensitivity has been explored in the literature, to the best of our knowledge no study has been made to investigate the impact of inter-scan variations. Thus, we investigated how noise in input data is transformed to noise in SPR and, by simulation, proton range for different dose and noise reduction levels, as well as for two different phantom sizes. To account for both intra- and inter-scan variations, 30 identical acquisitions were made for each investigated combination of experiment parameters.

## 2 Material and methods

### 2.1 Image acquisition

Image data was acquired on a GE Revolution CT (GE Healthcare, Waukesha, WI, USA) and a SOMATOM Definition Flash (Siemens Healthineers, Erlangen, Germany) using the inner (Head phantom) and a fully assembled (Body phantom) CIRS-062M phantom. The phantom inserts used in this work are given in Table S1. Two 4 cm thick slabs of water equivalent plastic were placed on each side of the phantom to ensure scatter equilibrium for the image acquisitions. Image data was acquired with a computed tomography dose index volume (CTDI_vol_) of 5, 10 and 15 mGy for the Body phantom, and 30, 40, and 50 mGy for the Head phantom.

On the GE scanner (Scanner 1) images were reconstructed with 2.5 mm slice thickness using the Standard reconstruction kernel with iterative noise reduction (ASiR-V) set to 0%, 40%, and 80%, or with deep learning-based noise reduction (True Fidelity) set to low, medium, and high. On the Siemens scanner (Scanner 2) VMI and native kV-images were reconstructed with 2.5 mm slice thickness using the QR40 kernel with iterative noise reduction (Safire) set to 0, 3, and 5. The two reconstruction kernels are comparable to each other with respect to image characteristics [20]. All image acquisitions were repeated 30 times.

The L-S method requires calibration, and to avoid calibrating the L-S method on the image data used for the analysis, one extra image acquisition was made for each phantom. These were acquired at medium dose level (10 mGy / 40 mGy CTDI_vol_), with no noise reduction.

While N&A requires no calibration, the accuracy depends on a DECT scanner’s ability to replicate accurate linear attenuation coefficients from VMI, so instead an optimization process may be used to determine the optimal VMI pairs for input with a given scanner (see Section S2 in the supplementary material). To investigate the robustness of this optimization process and avoid optimizing on the same image data used for the rest of the analysis (2.3, 2.4), an additional set of 30 identical image acquisitions were made for both phantoms at the medium dose level (10 mGy / 40 mGy CTDI_vol_) with no noise reduction.

### 2.2 SPR mapping methods

#### 2.2.1 Näsmark & Andersson

Jackson and Hawkes proposed the following parametrization of the linear attenuation coefficient μ,

(1) $$\mu \left(E\right)=\rho_{e}\left[Z_{\mathrm{eff}}^{4}F\left(Z_{\mathrm{eff}},E\right)+G\left(Z_{\mathrm{eff}},E\right)\right]$$

where $Z_{\mathrm{eff}}$ is the effective atomic number (EAN), $\rho_{e}$ is the relative electron density (RED), the term $Z_{\mathrm{eff}}^{4}F\left(Z_{\mathrm{eff}},E\right)$ represents photoelectric absorption and $G\left(Z_{\mathrm{eff}},E\right)$ represents both coherent and incoherent scattering [21]. Using two energies, $E_{1}$ and $E_{2}$, the following equations can be derived from Eq. (1),

(2) $$Z_{\mathrm{eff}}^{4}=\frac{\mu \left(E_{2}\right)G\left(Z_{\mathrm{eff}},E_{1}\right)-\mu \left(E_{1}\right)G\left(Z_{\mathrm{eff}},E_{2}\right)}{\mu \left(E_{1}\right)F\left(Z_{\mathrm{eff}},E_{2}\right)-\mu \left(E_{2}\right)F\left(Z_{\mathrm{eff}},E_{1}\right)},$$

(3) $$\rho_{e}=\frac{\mu \left(E_{1}\right)F\left(Z_{\mathrm{eff}},E_{2}\right)-\mu \left(E_{2}\right)F\left(Z_{\mathrm{eff}},E_{1}\right)}{F\left(Z_{\mathrm{eff}},E_{2}\right)G\left(Z_{\mathrm{eff}},E_{1}\right)-F\left({Z_{\mathrm{eff}},E}_{1}\right)G\left(Z_{\mathrm{eff}},E_{2}\right)}.$$

In this work, the SciPy package function Minimize (method=‘SLSQP’; x0 *=* 5; bounds = [4,54]) was used to solve Eq. (2) iteratively for the EAN. RED was calculated with Eq. (3) and mean ionization value (I-value) with the parametrization by Yang *et al*. [22],

(4) $$\ln(I)=\left\{\begin{array}{l} a_{1}∙Z_{\mathrm{eff}}+b_{1}Z_{\mathrm{eff}}<8.5\\ a_{2}∙Z_{\mathrm{eff}}+b_{2}Z_{\mathrm{eff}}\geq 8.5, \end{array}\right)$$

where the constants $a_{1}$ = 0.120, $a_{2}$=0.081, $b_{1}$=3.407, and $b_{2}$ = 3.514 were taken from our previous work [13]. Elemental I-values were taken from Bär *et al.* [23] when possible (H, C, N, O, P, Cl, Ca), or from ICRU report 37 (Na, Mg, S, K, Fe, I, Ba) [24].

Proton SPR was calculated using Bethe’s stopping power formula,

(5) $$\mathrm{SPR}=\rho_{e}\left[\left(\ln\frac{2m_{e}c^{2}\beta^{2}}{\left(1-\beta^{2}\right)I}-\beta^{2}\right)/\left(\ln\frac{2m_{e}c^{2}\beta^{2}}{\left(1-\beta^{2}\right)I_{w}}-\beta^{2}\right)\right]$$

where $m_{e}$ is the electron rest mass, c the speed of light in vacuum, β the proton speed relative to that of light in vacuum (using 100 MeV protons), and $I_{w}$ the I-value of water (set to 78.73 eV, based on the work by Bär *et al.)* [23].

The optimization process was repeated 30 times for each scanner to test for robustness against variations due to noise in input data. We investigated 5050 unique VMI pairs between 40 and 140 keV in steps of 1 keV and used a set of 73 theoretical reference tissues [26], [27], with linear attenuation coefficients taken from the NIST/XCOM database [25], to exclude VMI pairs that yielded SPR RMSE above 2%. The ground truth for the phantom inserts and the 73 reference tissues were calculated as described in Näsmark & Andersson (2021) [13]. For lung tissue, soft tissue, and bone, VMI pairs were ranked from the pair that yielded the lowest SPR RMSE to that which yielded the highest, and the optimal VMI pair was determined as the pair that yielded the lowest SPR RMSE across both phantoms.

The N&A method was applied on the image data described in Section 2.1 to generate EAN, RED and SPR maps using the optimal VMI pairs. On a voxel-to-voxel basis, the image volume was segmented based on the CT number in a reference VMI (74 keV) to determine which VMI pair to use as input. The segmentation limits (lung tissue: [<-200 HU], soft tissue: [-200, 150 HU], and bone: [>150 HU] were based on ROI measurements in reference VMIs from both scanners, for both phantom sizes, and using the medium dose level with no noise reduction.

#### 2.2.2 Landry-Saito

As described in Landry *et al*. [15], EAN can be determined with CT numbers from native low and high kV, $C{T\#}_{\mathrm{low}}$ and $\mathrm{CT}{\#}_{\mathrm{high}}$, images and the following equation,

(6) $$\frac{\mathrm{CT}{\#}_{low}/1000+1}{\mathrm{CT}{\#}_{high}/1000+1}=\frac{1+AZ_{\mathrm{eff}}^{m-1}}{B+CZ_{\mathrm{eff}}^{m-1}},$$

where the constants A,B,C, and m are obtained by least squares fitting of CT numbers from scans of phantom inserts with known EANs. Similarly, as presented in the work of Saito *et al*. [16], the RED can be determined from native low and high kV-images using the following equation,

(7) $$\rho_{e}=\frac{a\left[\left(1+\alpha \right)\mathrm{CT}{\#}_{high}-\alpha \mathrm{CT}{\#}_{low}\right]}{1000}+b,$$

where the constants α,a and b are obtained by least squares fitting of CT numbers and known REDs. The I-value and SPR can then be calculated with Eq. (4), (5), respectively.

In this work, separate calibrations were made for the two phantoms using the additional acquisitions described in Section 2.1. The SciPy package function curve_fit was used for the least square fittings, with initial guesses based on Table 2 in Hudobivnik *et al* [18].

**Table 1:** Optimal VMI pairs for SPR mapping using the N&A method on the two CT scanners used in this work, in how many iterations (out of 30) the VMI pair ranked first (out of 5050 investigated pairs), the mean SPR RMSE, standard deviation, and the lowest rank of the VMI pair across 30 iterations of the optimization process. Abbreviations: SPR = stopping power ratio; CT = computed tomography; VMI = virtual monoenergetic images; N&A = Näsmark & Andersson (2021); L-S = Landry-Saito.

	Tissue type	Optimal VMI Pair [keV]	Ranked first	SPR RMSE	Lowest ranking
Scanner 1	Lung tissue	113/114	20/30	5.0% ± 0.4%	11
	Soft tissue	49/52	10/30	0.5% ± 0.01%	7
	Bone	102/103	30/30	1.1% ± 0.03%	1

Scanner 2	Lung tissue	40/139	18/30	3.7% ± 1.3%	1792
	Soft tissue	44/54	9/30	0.4% ± 0.03%	13
	Bone	134/137	23/30	0.7% ± 0.3%	3

**Table 2:** Average 87% confidence interval (±1.5 standard deviations) and skewness (within parentheses) for the Breast insert at 10 mGy with different levels of iterative (ASiR-V / Safire) or deep-learning based (TF) noise reduction, measured in input data $\left(\mu /\mu_{H_{2}O}\right)$, EAN, RED and SPR maps generated with N&A, L-S, and DirectSPR methods for mapping SPR. No data is given for EAN and RED with DirectSPR, as the intermediary EAN and RED maps are not reconstructed. No data is given for EAN with L-S, as the EAN step is bypassed in our implementation of the method. Abbreviations: TF = True Fidelity; EAN = effective atomic number; RED = relative electron density; SPR = stopping power ratio; $\mu /\mu_{H_{2}O}$ = linear attenuation relative to water; N&A = Näsmark & Andersson (2021); L-S = Landry-Saito.

	Kernel and noise reduction	μlowμH2O	μhighμH2O	EAN	RED	SPR	Ranges
Scanner 1	Standard ASiR-V 0 (N&A)	±5,0% (0,01)	±4,6% (0,01)	±8,1% (-0,41)	±2,4% (0,02)	±1,8% (0,13)	±0,6% (0,28)
	Standard ASiR-V 40 (N&A)	±3,5% (0,01)	±3,2% (0,01)	±5,8% (-0,28)	±1,7% (0,01)	±1,3% (0,06)	±0,6% (0,19)
	Standard ASiR-V 80 (N&A)	±2,3% (0,01)	±2,1% (0,01)	±4,5% (-0,20)	±1,3% (<0,01)	±1,3% (<0,01)	±0,6% (0,23)
	Standard TF low (N&A)	±3,2% (0,05)	±3,0% (0,06)	±5,3% (-0,25)	±1,7% (0,05)	±1,3% (0,08)	±0,6% (0,36)
	Standard TF medium (N&A)	±2,7% (0,08)	±2,5% (0,08)	±4,6% (-0,22)	±1,5% (0,07)	±1,3% (0,06)	±0,7% (0,35)
	Standard TF high (N&A)	±2,1% (0,12)	±1,9% (0,13)	±4,2% (-0,19)	±1,4% (0,11)	±1,3% (0,10)	±0,7% (0,17)
Scanner 2	QR40 Safire 0 (N&A)	±8,2% (-0,01)	±5,9% (-0,01)	±14,2% (-0,65)	±2,9% (-0,03)	±1,9% (0,12)	±0,8% (0,31)
	QR40 Safire 3 (N&A)	±5,5% (<0,01)	±4,0% (<0,01)	±9,5% (-0,37)	±1,9% (-0,02)	±1,4% (0,04)	±0,7% (0,20)
	QR40 Safire 5 (N&A)	±3,9% (<0,01)	±2,7% (<0,01)	±6,9% (-0,23)	±1,3% (-0,01)	±1,0% (-0,02)	±0,6% (0,37)
	QR40 Safire 0 (L-S)	±5,0% (-0,01)	±4,8% (<0,01)	–	±10,1% (-0,01)	±10,2% (-0,01)	±1,5% (0,33)
	QR40 Safire 3 (L-S)	±3,3% (<0,01)	±3,2% (<0,01)	–	±6,7% (-0,01)	±6,8% (-0,01)	±1,2% (0,42)
	QR40 Safire 5 (L-S)	±2,2% (<0,01)	±2,1% (<0,01)	–	±4,5% (0,02)	±4,5% (0,02)	±1,2% (0,24)
	QR40 Safire 0 (DirectSPR)	±5,0% (-0,01)	±4,8% (<0,01)	–	–	±4,2% (0,03)	±1,0% (0,39)
	QR40 Safire 3 (DirectSPR)	±3,3% (<0,01)	±3,2% (<0,01)	–	–	±3,1% (0,07)	±0,9% (0,28)
	QR40 Safire 5 (DirectSPR)	±2,2% (<0,01)	±2,1% (<0,01)	–	–	±2,3% (0,08)	±0,9% (0,24)

The L-S method was applied to the native kV-image data described in Section 2.1. to generate RED and SPR maps. As the method produces unphysical (negative) EAN values for up to 25% of voxels in the input data (see Table S2), Eq. (4) could not be used for the EAN to I-value calculation on a pixel-to-pixel basis. Instead, the approach described by Zimmerman *et al.* was used to create a look-up table from $Z_{\mathrm{eff}}^{m-1}$ to ln(I) (Fig. S3) [28]. For a more comprehensive description and analysis of this approach, see Section S3.

#### 2.2.3 Siemens Healthineers DirectSPR

DirectSPR is a commercial application for generating proton SPR maps [17]. It is based on a method first proposed by Hünemohr *et al*. [7] where the RED and EAN are calculated voxel-wise by superimposing the CT numbers from the native low and high kV-images,

(8) $$\left(RED-1\right)∙1000HU=\alpha_{\mathrm{RED}}∙{\mathrm{CT}\#}_{\mathrm{low}}+\left(1-\alpha_{\mathrm{RED}}\right){\mathrm{CT}\#}_{\mathrm{high}}$$

(9) $${\mathrm{EAN}}^{3.1}=\frac{1}{\mathrm{RED}}\left[\alpha_{\mathrm{EAN}}\left(\frac{{\mathrm{CT}\#}_{\mathrm{low}}}{1000HU}+1\right)+\left({\mathrm{EAN}}_{\mathrm{Water}}^{3.1}-\alpha_{\mathrm{EAN}}\right)\left(\frac{{\mathrm{CT}\#}_{\mathrm{high}}}{1000HU}+1\right)\right],$$

where $\alpha_{\mathrm{RED}}$ and $\alpha_{\mathrm{EAN}}$ are calibration factors.

### 2.3 Image analysis

**Fig. 1:**
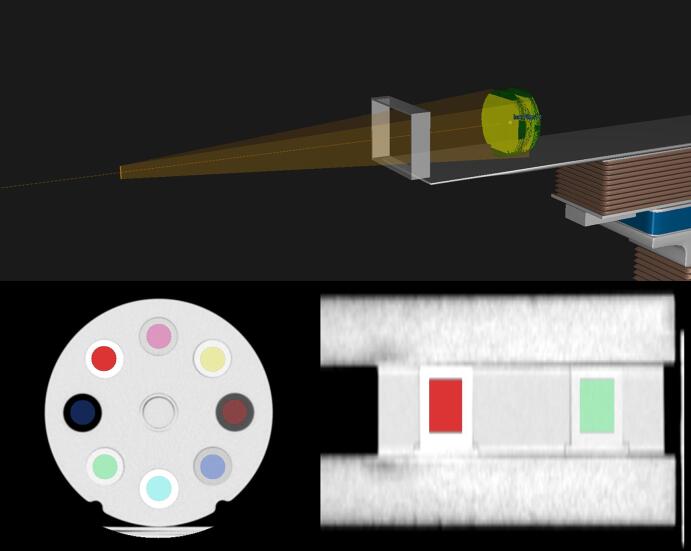
Top: treatment plan setup used for the simulations. Bottom left: axial view of ROI placement in phantom. Bottom right: sagittal view of ROI placement in phantom. Abbreviations: ROI = region of interest.

Sample mean, relative standard deviation and skewness were extracted with the image analysis software MICE Toolkit v2022.4.9 (Medical Interactive Creative Environment) using cylindrical (d = 20 mm, z = 30 mm) region of interests (ROI) at the centre of the phantom inserts (see Fig. 1) and averaged over each set of 30 acquisitions [29].

Due to the order of the Hounsfield unit (HU) scale (e.g., grayscale values: −1000 HU, +3000 HU), relative standard deviations would be inflated for CT numbers in soft tissue materials (∼0 HU) in VMIs and native kV-images. To avoid this, CT numbers were expressed as linear attenuation relative to water,

(10) $$\frac{\mu_{x}}{\mu_{H_{2}O}}=\frac{\left(CT\#+1000\right)}{1000}.$$

### 2.4 Proton range simulation

Treatment plans were created in RayStation v12.0.130.97 (RaySearch Laboratories, Stockholm, Sweden) based on SPR maps generated with the three investigated methods. For the simulations, a 105 MeV beam was applied perpendicular to the face of the phantom (see Fig. 1) and the dose grid resolution was set to 2x2x1 mm. To simplify measurements of simulated range, incident proton tracks were set parallel to each other. For more details, see Table S5.

The measurement point of simulated proton range was defined as the distance to the distal 80% dose drop off and measured in the dose grid along 81 lines passing through a circular ROI (d = 20 mm) at the centre of the phantom inserts. Mean range, standard deviation, and skewness were calculated for each ROI and averaged over each set of 30 acquisitions.

## 3 Results

### 3.1 Optimization process robustness (N&A)

**Fig. 2:**
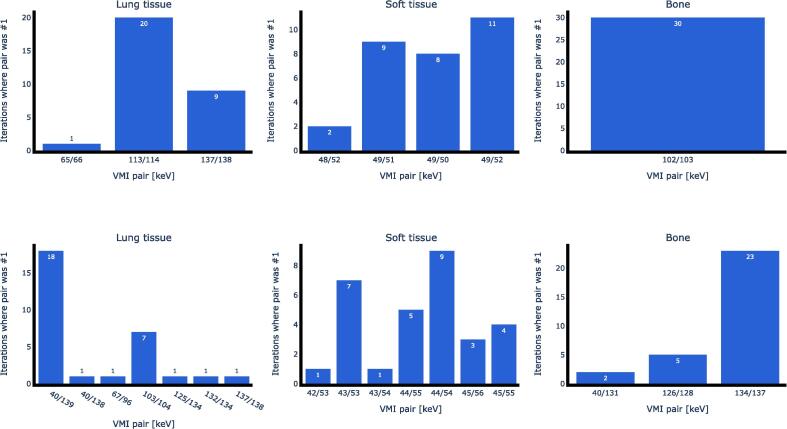
How many iterations of the optimization process (out of 30) that yielded a given optimal VMI pair for SPR mapping soft tissue using the N&A method on Scanner 1 (top row) and Scanner 2 (bottom row). Abbreviations: VMI = virtual monoenergetic images; SPR = stopping power ratio; N&A = Näsmark & Andersson (2021).

Table 1 shows the results for 30 optimization process iterations of the N&A method. The two scanners show similar SPR RMSE for soft tissue, while VMIs from Scanner 2 perform better for lung tissue and bone. Low energy VMI pairs works best for soft tissue, while higher energies work best for bone. The 30 best scoring VMI pairs for soft tissue are clustered close together (Fig. 2). The optimal VMI pairs in general perform well even in iterations where they were not the top performing pairs (Table 1). At worst they still rank within the top 0.3% (out of 5050), the only exception being Lung tissue on Scanner 2 where the overall optimal pair ranks within the top 0.3% in 20 iterations and within the top 29–36% pairs in the remaining 10 iterations. While mean SPR RMSEs are comparable between scanners, standard deviations are one order of magnitude larger on Scanner 2 for lung tissue and bone.

### 3.2 Noise analysis from ROI statistics and proton range estimations

Table 2, Table 3 show examples of how noise in the input data is transformed to noise in the generated EAN, RED and SPR images, and simulated proton range. Behaviours observed in these two inserts, Breast and Bone 800, are representative for the soft tissue and bone inserts, respectively. As can be seen, noise in input data is amplified with L-S and kept level with DirectSPR. While N&A in general compresses noise, we found that for some VMI pairs the EAN distribution collapses into discrete spikes for bone, which in turn increases noise in the SPR images (Table 3). This is further explored in Section S5. There was also a minor trend in input data where skewness increased with noise reduction and dose level on both scanners.

**Table 3:** Average 87% confidence interval (±1.5 standard deviations) and skewness (within parentheses) for the Bone 800 insert at 10 mGy with different levels of iterative (ASiR-V / Safire) or deep-learning based (TF) noise reduction, measured in input data $\left(\mu /\mu_{H_{2}O}\right)$, EAN, RED and SPR maps generated with N&A, L-S, and DirectSPR methods for mapping SPR. No data is given for EAN and RED with DirectSPR, as the intermediary EAN and RED maps are not reconstructed. No data is given for EAN with L-S, as the EAN step is bypassed in our implementation of the method. Abbreviations: TF = True Fidelity; EAN = effective atomic number; RED = relative electron density; SPR = stopping power ratio; $\mu /\mu_{H_{2}O}$ = linear attenuation relative to water; N&A = Näsmark & Andersson (2021); L-S = Landry-Saito.

	Kernel and noise reduction	μlowμH2O	μhighμH2O	EAN	RED	SPR	Ranges
Scanner 1	Standard ASiR-V 0 (N&A)	±1,5% (-0,03)	±1,5% (-0,03)	±5,2% (0,73)	±2,1% (-0,12)	±2,6% (-0,24)	±0,4% (0,35)
	Standard ASiR-V 40 (N&A)	±1,2% (-0,02)	±1,2% (-0,02)	±5,5% (0,60)	±2,0% (-0,36)	±2,6% (-0,43)	±0,3% (0,37)
	Standard ASiR-V 80 (N&A)	±0,9% (-0,01)	±0,9% (-0,01)	±4,9% (1,51)	±1,8% (-0,74)	±2,3% (-0,95)	±0,3% (0,19)
	Standard TF low (N&A)	±1,1% (0,02)	±1,1% (0,02)	±5,6% (<0.01)	±2,0% (-0,29)	±2,6% (-0,27)	±0,4% (0,51)
	Standard TF medium (N&A)	±0,9% (0,04)	±0,9% (0,04)	±5,3% (0,75)	±1,9% (-0,49)	±2,4% (-0,58)	±0,4% (0,33)
	Standard TF high (N&A)	±0,8% (0,08)	±0,8% (0,08)	±5,1% (1,21)	±1,7% (-0,72)	±2,3% (-0,86)	±0,4% (0,47)
Scanner 2	QR40 Safire 0 (N&A)	±2,3% ((<0.01)	±2,4% (<0.01)	±7,1% (-0,54)	±3,1% (0,02)	±3,7% (0,08)	±0,7% (0,55)
	QR40 Safire 3 (N&A)	±1,6% ((<0.01)	±1,7% (<0.01)	±6,1% (-0,46)	±2,3% (0,04)	±2,8% (0,13)	±0,7% (0,25)
	QR40 Safire 5 (N&A)	±1,2% (-0,01)	±1,2% (-0,01)	±5,6% (-0,71)	±1,8% (0,08)	±2,3% (0,26)	±0,6% (0,48)
	QR40 Safire 0 (L-S)	±3,1% (-0,02)	±3,3% (<0.01)	–	±8,8% (0,01)	±8,9% (0,01)	±0,9% (0,38)
	QR40 Safire 3 (L-S)	±2,3% (-0,03)	±2,2% (-0,01)	–	±6,2% (0,01)	±6,2% (0,01)	±0,8% (0,35)
	QR40 Safire 5 (L-S)	±1,8% (-0,08)	±1,6% (-0,02)	–	±4,5% (0,01)	±4,5% (0,01)	±0,8% (0,38)
	QR40 Safire 0 (DirectSPR)	±3,1% (-0,02)	±3,3% (<0.01)	–	–	±3,0% (-0,01)	±0,8% (0,39)
	QR40 Safire 3 (DirectSPR)	±2,3% (-0,03)	±2,2% (-0,01)	–	–	±2,3% (-0,01)	±0,8% (0,16)
	QR40 Safire 5 (DirectSPR)	±1,8% (-0,08)	±1,6% (-0,02)	–	–	±1,8% (-0,06)	±0,6% (0,25)

In general, range uncertainty was lower for N&A than for the two kV-based methods. For the body phantom, range uncertainty with N&A was lower on Scanner 1 compared to Scanner 2. While reducing noise in the input data reduced noise in the SPR maps with up to 1.5%, uncertainty in proton range was reduced at most with 0.4%.

Increasing CTDI_vol_ from 5 mGy to 15 mGy reduced range uncertainty by up to ∼0.4% in the Body phantom. For the Head phantom, increasing from 30 to 50 mGy reduced the uncertainty by ∼0.04% with N&A and ∼0.8% with L-S. Increasing iterative or deep-learning based noise reduction reduced the uncertainty with up to 0.04% on Scanner 1 and up to 0.2% on Scanner 2.

Proton SPR and range for all investigated settings are given in Tables S7 - S18.

## 4 Discussion

In this study, we have evaluated the noise-sensitivity of three DECT-based algorithms for SPR mapping. We found that noise in input data was amplified in SPR maps generated with L-S, kept level with DirectSPR, and compressed with N&A. In general, range uncertainty was lower with N&A than with L-S and DirectSPR.

Range uncertainties averaged over tissue type with N&A on Scanner 1 were, except for at 5 mGy CTDI_vol_, smaller than the 0.5% range uncertainty contribution attributed to CT image noise in Paganetti *et al* [5]. On Scanner 2, uncertainties were larger than 0.5% for all three methods. However, the 0.5% contribution is based on a theoretical scenario where range is estimated in a perfectly homogeneous material with added gaussian noise (2.5%) and 3x3x3 mm voxel size [30]. The uncertainties presented here are based on CT image data, estimated in materials that are not perfectly homogeneous, and simulated with 2x2x1 mm voxel size.

Lee *et al.* reported range uncertainties due to 2% DECT image noise within 0.9% and 0.8% (presented as 95% confidence intervals in original publication, converted here to 87%) for soft tissue and bone, respectively [31]. Averaged over tissue type, only N&A on Scanner 1 consistently comes within these values (see Tables S13-S18). Bär *et al.* showed that DECT SPR mapping methods (using kV-images as input) may lose their advantage over the conventional method at these noise levels [3]. Our results support the conclusions of these previous works, that while range uncertainty due to noise in general is small compared to other contributions, it becomes more important as we move towards smaller treatment margins and the noise-sensitivity of SPR mapping methods should be carefully estimated and considered before clinical implementation.

VMIs are reconstructed in projection space on Scanner 1 and image space on Scanner 2 [32]. Wohlfahrt *et al.* showed that when VMIs are reconstructed in image space, there is a steep increase in image noise for energies below 70 keV [33]. This effect can be seen in Table 2, Table 3, as VMIs on Scanner 2 are noisier than native kV-images for soft tissue (44/54 keV) but not for bone (134/137 keV).

The energy separation for all optimal VMI pairs, except one, is just a few keV (Table 1). This may appear counterintuitive, but all VMIs are generated from the same low and high kV scan data and in theory a majority of the investigated VMI pairs (89% for lung tissue, 93% for soft tissue, and 79% for Bone) result in a SPR RMSE within 1% (see Fig. S1 and S2). This indicates that optimal VMI pairs are a result of the imaging chain rather than the N&A method itself, and that low energy separation in VMI pairs is a consequence of linear attenuation coefficients derived from VMI yielding the most accurate SPR.

Since some VMI pairs may result in collapsed EAN distributions and amplified SPR noise (see Section S5), normally distributed EAN should be added as an additional condition in the optimization process. This is not a consequence of the method itself, as a majority of the possible VMI pairs work in theory (Fig. S1 and S2), but rather a reality of the imaging chain.

A limitation of this study was that there was no ground truth to evaluate the estimated proton ranges. Further investigations including physical measurements on proton SPR and range could give better insights into precision and uncertainty of the methods analysed in the present work. Alternatively, or additionally, a virtual model of the setup could be built in a treatment planning system. However, a problem with virtual models is the ground truth uncertainty associated with the elemental composition of tissue equivalent inserts. Both approaches were out of scope for the present study, given the number of physical measurements performed, and image data acquired for analysis.

From results presented in this and our previous work, we believe that the N&A method is ready for preclinical evaluation and translation. In preclinical evaluation, the method should be studied and verified, e.g., on animal tissue and through retrospective dose studies and a wider uncertainty analysis that takes all clinically relevant contributions into account. While DirectSPR is already commercially available and clinically verified [25], the N&A method warrants further studies as it is firmly based on well-established x-ray physics formalism, is CT-vendor neutral and, thus is directly applicable with CT scanners which cannot produce high and low kV-images [13].

## Funding information

Barncancerfonden (Swedish Childhood Cancer Foundation), Grant/Award Number: MT2018-0014, MT2020-0011.

Cancerforskningsfonden i Norrland/Lions Cancerforskningsfond i Norr, Grant/Award Number: AMP-1099.

## CRediT authorship contribution statement

**Torbjörn Näsmark:** Conceptualization, Methodology, Software, Validation, Formal analysis, Investigation, Data curation, Writing – original draft, Writing – review & editing, Visualization, Project administration, Funding acquisition. **Jonas Andersson:** Conceptualization, Methodology, Formal analysis, Writing – review & editing, Supervision, Project administration, Funding acquisition.

## Declaration of Competing Interest

The authors declare that they have no known competing financial interests or personal relationships that could have appeared to influence the work reported in this paper.
