# Supplementary material for: The influence of dual-energy computed tomography image noise in proton therapy treatment planning
Source: Phys Imaging Radiat Oncol. 2023 Sep 20;28:100493. doi: 10.1016/j.phro.2023.100493 (PMC10544042; doi:10.1016/j.phro.2023.100493)
Supplement: Supplementary data 1 [file mmc1.docx]

**Supplemental material to: The Influence of Dual-Energy Computed Tomography Image Noise in Proton Therapy Treatment Planning**

**Section S1. CIRS 062M phantom tissue equivalent inserts**

Table 1 contains a list of the CIRS 062M phantom inserts used in this work.

Table S1. CIRS 062M inserts used in this work.

| **Insert** |
| --- |
| Lung (inhale) |
| Lung (exhale) |
| Adipose |
| Breast |
| Liver |
| Muscle |
| Bone 200 |
| Bone 800 |

**Section S2. Optimization process for the N&A method**

The Näsmark & Andersson (2021) method (N&A) requires no calibration, but the accuracy depends on a DECT scanner’s ability to replicate true monoenergetic images, so instead an optimization process may be used to determine the optimal VMI pairs for input with a given scanner [13]. This optimization process consists of the following steps:

1. Scan tissue equivalent phantom inserts of known composition and calculate the SPR root mean square error (RMSE) for each possible VMI pair.
2. To ensure compliance with theory, calculate SPR for a set of theoretical reference tissues [26, 27] and exclude VMI pairs that yield SPR RMSEs exceeding a suitable limit.
3. Select the VMI pair that yields the lowest SPR RMSE.

In Näsmark and Andersson (2021), this optimization yielded VMI pairs with an accuracy around 0.5% for soft tissue and 1% for bone, while most other VMI pairs had an accuracy of 1-2% for soft tissue and 2-6% for bone [13]. As can be seen in Fig. S1 and S2, the optimizations performed in this work behave in the same way and support the conclusion that the accuracy depends on VMI authenticity. In theory, a majority of the investigated VMI pairs (89% for lung tissue, 93% for soft tissue, and 79% for Bone) result in a SPR RMSE within 1%.

The choice of 74 keV as the reference VMI for the segmentation described in Section 2.2.1 was selected as it should be close to the mean energy of a common 120 kV-image CT scan. It is possible that another keV would be more suitable, and there are other segmentation methods with more finesse. However, further investigation into this was considered outside the scope of this work.

While the optimization process for the N&A method seems to work satisfactory, using multiple optimizations depending on the patient size is probably a sound strategy. As an example, the optimal VMI pair for bone locks in on 102/103 keV, which is the best performing VMI pair for bone in the Head phantom with a SPR RMSE of 0.8%. However, by choosing that VMI pair for both phantoms, the SPR RMSE for the Body phantom ends up being 1.4%. Had we instead applied the optimization process on each phantom separately, we would have ended up with 96/97 keV and a SPR RMSE of 0.1%. According to Peters *et al*., a size-dependent calibration is already incorporated in DirectSPR [17].


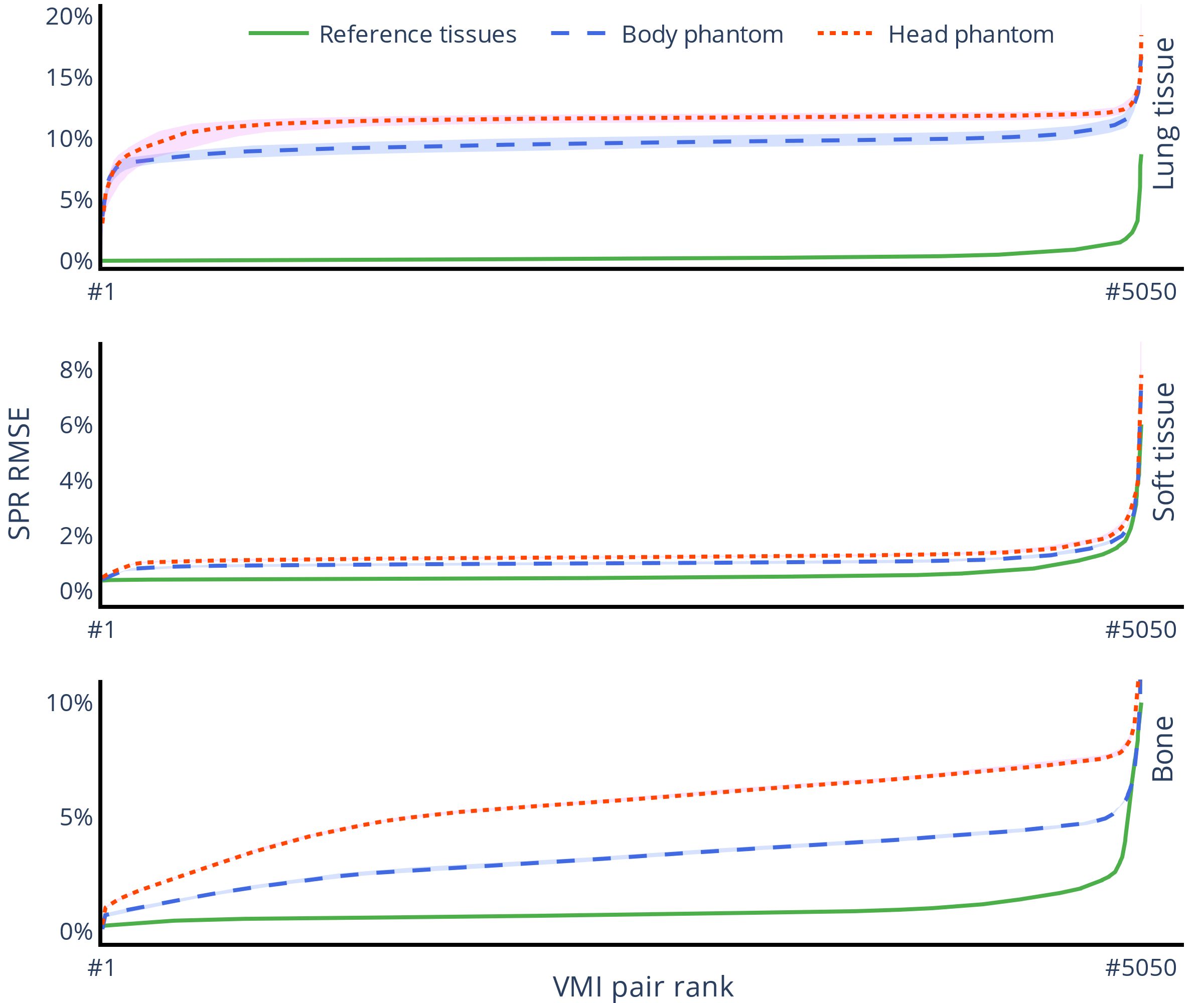


Figure S1. SPR RMSE plotted against VMI pair rank (1 to 5050), for 30 optimizations on Scanner 1. The opaque lines show mean SPR RMSE for the 30 optimizations, while the shaded areas show minimum and maximum values. Abbreviations: SPR = stopping power ratios; RMSE = root mean square error; VMI = virtual monoenergetic images.


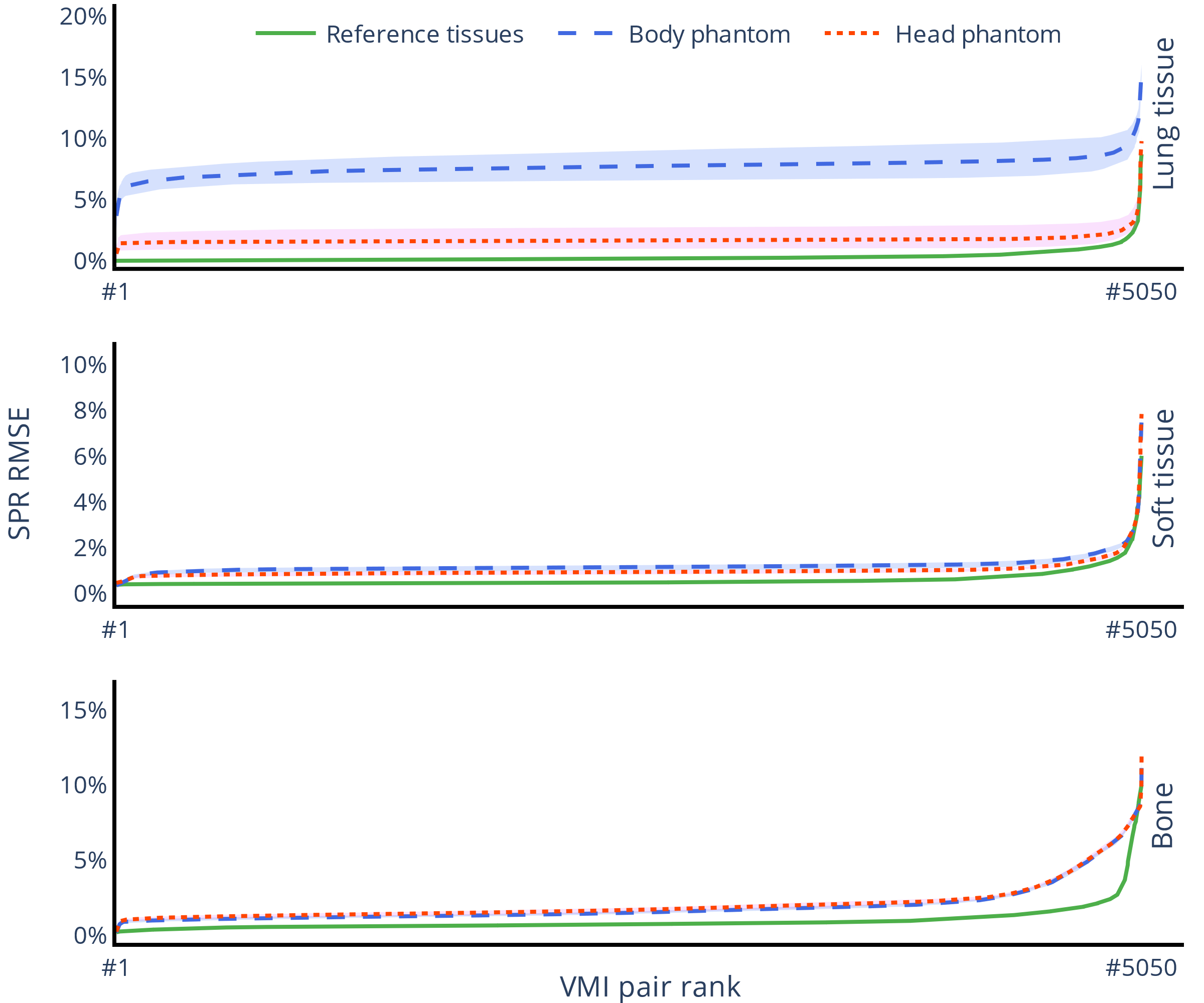


Figure S2. SPR RMSE plotted against VMI pair rank (1 to 5050), for 30 optimizations on Scanner 2. The opaque lines show mean SPR RMSE for the 30 optimizations, while the shaded areas show minimum and maximum values. Abbreviations: SPR = stopping power ratios; RMSE = root mean square error; VMI = virtual monoenergetic images.

**Section S3. Unphysical EAN with the Landry-Saito method**

As the Landry-Saito (L-S) method [15, 16] produces unphysical (negative) EAN values for some pixels in input data, we could not use Eq. (4) for the EAN to I-value calculation on a pixel-to-pixel basis. Instead, we used the approach described by Zimmerman *et al.* [35] and created a look-up table from $Z_{eff}^{m-1}$to $\ln(I)$ (Fig. S3). Table S2 shows the ratio of voxels in the EAN image volume with unphysical values.


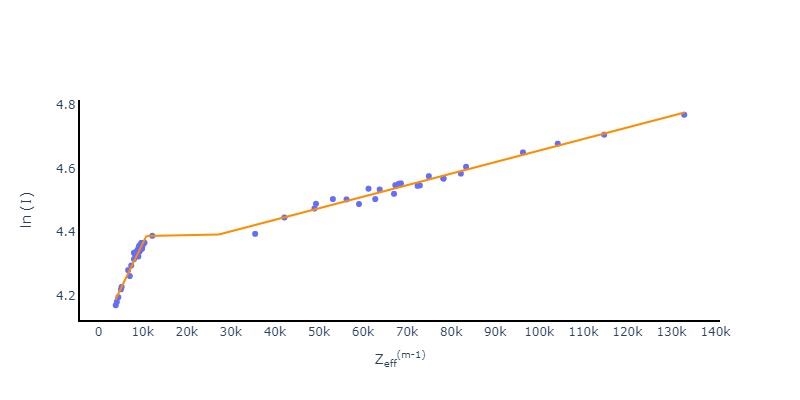


Figure S3. Scatter plot of $Z_{eff}^{(m-1)}$ against $\ln(I)$ for 72 theoretical reference tissues. The line shows the piece-wise linear fit used to estimate $\ln(I)$ for the stopping power ratio calculations. Abbreviations: k = 1000; Z_eff_ = effective atomic number; $\ln(I)$ = natural logarithm of mean ionization value.

Table S2. The ratio of voxels in the image volume that end up with unphysical EANs due to image noise in the native low and high kV-images, for different levels of iterative noise reduction (Safire). Unphysical EANs in the air outside the phantom have been excluded from the count. Abbreviations: EAN = effective atomic number; CTDI_vol_ = computed tomography dose index.

| **CTDIvol** | **Safire** | **Body phantom** | **Head phantom** |
| --- | --- | --- | --- |
| 5 mGy | 0 | 23,8 % | 25,2 % |
|  | 3 | 19,0 % | 23,5 % |
|  | 5 | 15,5 % | 22,7 % |
|  |  |  |  |
| 10 mGy | 0 | 23,4 % | 24,8 % |
|  | 3 | 19,2 % | 23,5 % |
|  | 5 | 15,8 % | 21,9 % |
|  |  |  |  |
| 15 mGy | 0 | 22,7 % | 24,1 % |
|  | 3 | 18,8 % | 22,0 % |
|  | 5 | 15,0 % | 21,3 % |

The potential impact of this approach was investigated by comparing the accuracy of the Zimmerman *et al*. [28] approach against the parametrization, Eq. (3), by Yang *et al.* [22] in the Body (CTDIvol = 10 mGy, Safire 0) and the Head phantom (CTDIvol = 40 mGy, Safire 0). The relative mean deviation from ground truth was measured in $ln(I)$ and $SPR$ maps by placing circular ROIs (d = 1 cm) in the centre of the phantom inserts. For the $ln(I)$ calculations with Eq. (3), voxels with negative EAN values were set to Not a Number.

In Näsmark & Anderson (2021), the uncertainty in SPR ground truth $\sigma_{SPR}$ due to uncertainties in mass density given by the manufacturer, elemental I-values and the I-value of water was estimated to be around 1% [13]. As can be seen in Table S3 (Head phantom) and S4 (Body phantom), relative SPR deviations from ground truth are within two $\sigma_{SPR}$ from each other except for all inserts except Lung (inhale) and Bone 800 in the Body phantom.

*Table S3.* Relative $\ln\left( I \right)$ and SPR deviation from ground truth using the approach from Zimmerman *et al*. compared to the parametrization of Yang *et al*. Abbreviations: $\ln\left( I \right)$ = natural logarithm of mean ionization values; SPR = relative stopping power.

|  | $\ln\left( \boldsymbol{I} \right)$ | |  |  | **SPR** | |
| --- | --- | --- | --- | --- | --- | --- |
| **Insert** | **Zimmerman** | **Yang** |  |  | **Zimmerman** | **Yang** |
| Lung (inhale) | 0,9% | 0,7% |  |  | -4,6% | 5,6% |
| Lung (exhale) | 1,3% | 1,1% |  |  | -1,1% | 1,0% |
| Adipose | -0,9% | 2,5% |  |  | 1,3% | 2,0% |
| Breast | -0,2% | 0,9% |  |  | 0,7% | 1,1% |
| Liver | 1,2% | 1,0% |  |  | -2,8% | 2,7% |
| Muscle | 1,3% | 1,1% |  |  | -0,1% | 0,1% |
| Bone 200 | 0,3% | 1,2% |  |  | -2,1% | 1,3% |
| Bone 800 | 1,8% | 1,1% |  |  | -0,5% | 0,1% |

Table S4. Relative $\ln\left( I \right)$ deviation from ground truth using the approach from Zimmerman et al. compared to the parametrization of Yang et al. Abbreviations: $\ln\left( I \right)$ = natural logarithm of mean ionization values; SPR = relative stopping power.

|  | $\ln\left( \boldsymbol{I} \right)$ | |  | **SPR** | |
| --- | --- | --- | --- | --- | --- |
| **Insert** | **Zimmerman** | **Yang** |  | **Zimmerman** | **Yang** |
| Lung (inhale) | -3,6% | 2,1% |  | -7,1% | 28,6% |
| Lung (exhale) | -3,9% | 1,9% |  | 0,2% | -5,3% |
| Adipose | -2,8% | 2,9% |  | 1,9% | -0,1% |
| Breast | -3,1% | 3,4% |  | 2,6% | 1,3% |
| Liver | -3,7% | 2,6% |  | 1,4% | 0,1% |
| Muscle | -3,7% | 2,7% |  | 2,4% | 0,9% |
| Bone 200 | -7,1% | 2,0% |  | 2,0% | -0,7% |
| Bone 800 | -11,1% | 5,0% |  | 6,6% | 3,0% |

**Section S4. RayStation parameters**

Table S5 shows the parameter settings that were used for the simulations in RayStation. The dose calculations were made using Monte Carlo with the uncertainty level set to 0.5%.

*Table S5.* RayStation parameter settings.

| **Parameter** | **Set to** |
| --- | --- |
| Treatment technique | Pencil Beam Scanning |
| Treatment head | RSL_PBS_CYCL_PHY |
| Fraction dose | 1 |
| RBE model | constant 1.1 |
| Beam set comment field | “/MCOPT=B9” ^1^ |
| Dose grid resolution | 2 mm x 2 mm 1 x mm |
| Snout | snout40 |
| Beam isocentre | Centre of the External ROI |
| MU/fx | 100 |
| External HU threshold | 90 |
| Nominal Energy | 105 MeV |
| spot spacing | 0.2 cm |
| spot pattern | square |
| energy layer | width and height both set to 18 cm for the head phantom and 30 cm for the Body phantom. |

*^1^* A special command making all incident proton tracks parallel to each other.

**Section S5. VMI pair energy separation and noise compression**

N&A compresses noise for all tissue types. However, we found that for some VMI pairs the EAN distribution collapses to discrete spikes for bone, which in turn increases noise in the EAN, RED and SPR images (Fig. S4 and S6). Increasing the VMI pair energy separation alleviated this issue (Table S6, Fig. S5 and S7), but it remains unclear what role this actually plays as a narrow separation works well for soft tissue inserts in the CIRS phantom, see, e.g., propagation of noise in Table 2 for the Breast insert.

Thus, for optimization of the N&A method, it is advised to require that EAN must be normally distributed as an extra condition in the optimization process for choosing the optimal VMIs. As a discrete spike in the EAN distribution may pass the skewness criteria (within ±0.5), visual inspection of distributions or another more comprehensive test is required to exclude collapsed EAN distributions in the optimization process.

Table S6. Average 87% confidence interval (±2 standard deviations) and skewness (within parentheses) for the Bone 800 insert at 10 mGy CTDI_vol_ with no noise reduction, with different VMI pairs used as input, measured in input data $\boldsymbol{\mu}/{\boldsymbol{\mu}_{\boldsymbol{H}_{\boldsymbol{2}}\boldsymbol{O}}}$EAN, RED and SPR maps generated with N&A [13]. Abbreviations: CTDI_vol_ = computed tomography dose index; VMI = virtual monoenergetic images; $\boldsymbol{\mu}/{\boldsymbol{\mu}_{\boldsymbol{H}_{\boldsymbol{2}}\boldsymbol{O}}}$=linear attenuation relative to water; EAN = effective atomic number; RED = relative electron density; SPR = stopping power ratios; N&A = Näsmark & Andersson (2021).

|  | **VMI pair** | $\frac{\boldsymbol{\mu}_{\boldsymbol{low}}}{\boldsymbol{\mu}_{\boldsymbol{H}_{\boldsymbol{2}}\boldsymbol{O}}}$ | $\frac{\boldsymbol{\mu}_{\boldsymbol{high}}}{\boldsymbol{\mu}_{\boldsymbol{H}_{\boldsymbol{2}}\boldsymbol{O}}}$ | **EAN** | **RED** | **SPR** |
| --- | --- | --- | --- | --- | --- | --- |
| **Scanner 1** | 102/103 keV | ±1,5% (-0,03) | ±1,5% (-0,03) | ±5,2% (0,73) | ±2,1% (-0,12) | ±2,6% (-0,24) |
|  | 49/114 keV | ±2,1% (-0,02) | ±1,4% (-0,03) | ±0,5% (0,12) | ±1,3% (-0,03) | ±1,2% (-0,03) |
|  |  |  |  |  |  |  |
| **Scanner 2** | 134/137 keV | ±2,3% (0,00) | ±2,4% (<0.01) | ±7,1% (-0,54) | ±3,1% (0,02) | ±3,7% (0,08) |
|  | 40/139 keV | ±5,1% (0,03) | ±2,4% (0,02) | ±2,3% (-0,05) | ±2,4% (<0,01) | ±2,5% (0,01) |


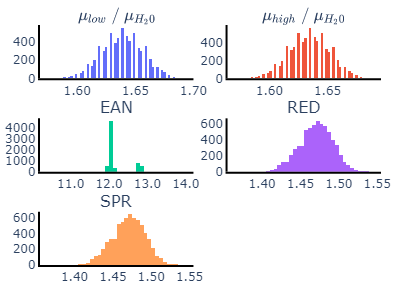


Figure S4. Histograms for the Bone 800 insert in input data $\left( \boldsymbol{\mu}/{\boldsymbol{\mu}_{\boldsymbol{H}_{\boldsymbol{2}}\boldsymbol{O}}} \right)$, EAN, RED and SPR maps generated with N&A [13] using VMI pair 102/103 used as input on Scanner 1. Abbreviations: $\boldsymbol{\mu}/{\boldsymbol{\mu}_{\boldsymbol{H}_{\boldsymbol{2}}\boldsymbol{O}}}$ = linear attenuation relative to water; EAN = effective atomic number; RED = relative electron density; SPR = stopping power ratios; N&A = Näsmark & Andersson (2021); VMI = virtual monoenergetic images.


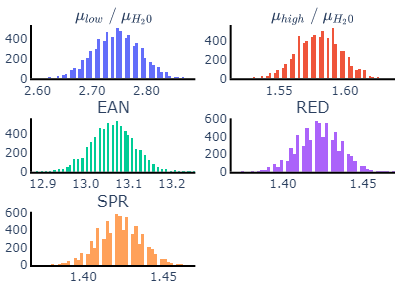


Figure S5. Histograms for the Bone 800 insert in input data $\boldsymbol{\mu}/{\boldsymbol{\mu}_{\boldsymbol{H}_{\boldsymbol{2}}\boldsymbol{O}}}$, EAN, RED and SPR maps generated with N&A [13] using VMI pair 49/114 used as input on Scanner 1. Abbreviations: $\boldsymbol{\mu}/{\boldsymbol{\mu}_{\boldsymbol{H}_{\boldsymbol{2}}\boldsymbol{O}}}$ = linear attenuation relative to water; EAN = effective atomic number; RED = relative electron density; SPR = stopping power ratios; N&A = Näsmark & Andersson (2021); VMI = virtual monoenergetic images.


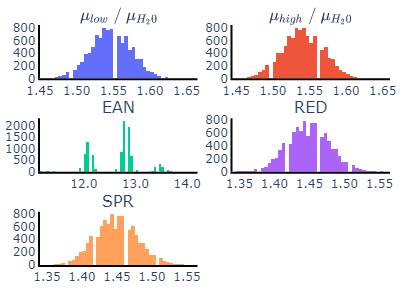


Figure S6. Histograms for the Bone 800 insert in input data $\left( \boldsymbol{\mu}/{\boldsymbol{\mu}_{\boldsymbol{H}_{\boldsymbol{2}}\boldsymbol{O}}} \right)$, EAN, RED and SPR maps generated with N&A [13] using VMI pair 134/137 used as input on Scanner 2. Abbreviations: $\boldsymbol{\mu}/{\boldsymbol{\mu}_{\boldsymbol{H}_{\boldsymbol{2}}\boldsymbol{O}}}$ = linear attenuation relative to water; EAN = effective atomic number; RED = relative electron density; SPR = stopping power ratios; N&A = Näsmark & Andersson (2021); VMI = virtual monoenergetic images.


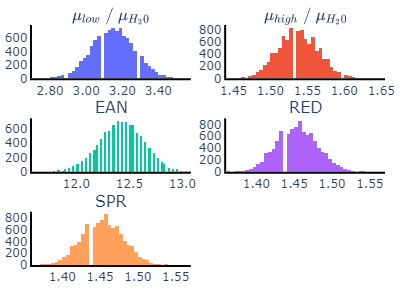


Figure S7. Histograms for the Bone 800 insert in input data $\left( \boldsymbol{\mu}/{\boldsymbol{\mu}_{\boldsymbol{H}_{\boldsymbol{2}}\boldsymbol{O}}} \right)$, EAN, RED and SPR maps generated with N&A [13] using VMI pair 40/139 used as input on Scanner 2. Abbreviations: $\boldsymbol{\mu}/{\boldsymbol{\mu}_{\boldsymbol{H}_{\boldsymbol{2}}\boldsymbol{O}}}$ = linear attenuation relative to water; EAN = effective atomic number; RED = relative electron density; SPR = stopping power ratios; N&A = Näsmark & Andersson (2021); VMI = virtual monoenergetic images.

**Section S6. SPR tables**

Tables S7-S12 shows the mean SPR with 87% confidence intervals (± 1.5 standard deviations) and skewness (within parenthesis) measured in the SPR images generated with N&A [13], L-S [15,16] and DirectSPR [17]. The SPR images were generated from image data acquired with 30, 40, 50 mGy CTDI_vol_ for the head phantom and 5, 10, and 15 mGy CTDI_vol_ for the Body phantom, with different levels of iterative or deep-learning based noise reduction. To account for intra- and inter-scan variabilities, the mean values and confidence intervals have been averaged over 30 identical acquisitions.

Table S7. Mean SPR and 87% confidence intervals (± 1.5 SD) and skewness (within parenthesis) measured in SPR images generated with N&A [13] and L-S [15,16] from image data acquired at 30 mGy CTDI_vol_ with different levels of iterative noise reduction (ASiR-V / Safire). Abbreviations: SPR = stopping power ratios; SD = standard deviation; N&A = Näsmark & Andersson (2021); L-S = Landry-Saito; CTDI_vol_ = computed tomography dose index.

| Scanner 2 Scanner 1  Scanner 2 | **Kernel and noise reduction** | **Lung (inhale)** | **Lung (exhale)** | **Adipose** | **Breast** | **Liver** | | | **Muscle** | **Bone 200** | | | **Bone 800** |  |
| --- | --- | --- | --- | --- | --- | --- | --- | --- | --- | --- | --- | --- | --- | --- |
|  | Standard ASiR-V 0 (N&A) | 0.22 ± 11.8 % (1,88) | 0.51 ± 3.4 % (1,27) | 0.97 ± 1.2 % (0,25) | 1.00 ± 0.9 % (0,03) | | 1.06 ± 0.9 % (0,11) | 1.06 ± 1.1 % (0,21) | | | 1.10 ± 3.3 % (-1,4) | 1.41 ± 2.8 % (0,14) | |  |
|  | Standard ASiR-V 40 (N&A) | 0.22 ± 11.7 % (1,95) | 0.51 ± 3.1 % (1,94) | 0.97 ± 1.0 % (0,45) | 1.00 ± 0.7 % (0,08) | | 1.06 ± 0.7 % (0,18) | 1.06 ± 0.9 % (0,37) | | | 1.10 ± 3.3 % (-1,47) | 1.41 ± 2.8 % (0,14) | |  |
|  | Standard ASiR-V 80 (N&A) | 0.22 ± 11.7 % (2,01) | 0.51 ± 2.8 % (2,91) | 0.97 ± 0.9 % (0,55) | 1.00 ± 0.7 % (0,14) | | 1.06 ± 0.7 % (0,24) | 1.06 ± 0.8 % (0,48) | | | 1.10 ± 3.3 % (-1,51) | 1.41 ± 2.8 % (0,14) | |  |
|  |  |  |  |  |  | |  |  | | |  |  | |  |
|  | QR40 Safire 0 (N&A) | 0.20 ± 5.6 % (-0,03) | 0.50 ± 1.7 % (0,01) | 0.97 ± 0.6 % (0,05) | 1.00 ± 0.6 % (-0,08) | | 1.06 ± 0.6 % (-0,01) | 1.06 ± 0.6 % (<0,01) | | | 1.10 ± 1.0 % (0,14) | 1.41 ± 1.6 % (0,74) | |  |
|  | QR40 Safire 3 (N&A) | 0.20 ± 4.8 % (-0,05) | 0.50 ± 1.4 % (0,11) | 0.97 ± 0.5 % (0,11) | 1.00 ± 0.5 % (-0,09) | | 1.06 ± 0.4 % (0,03) | 1.06 ± 0.5 % (-0,07) | | | 1.10 ± 0.8 % (0,29) | 1.41 ± 1.4 % (1,06) | |  |
|  | QR40 Safire 5 (N&A) | 0.20 ± 4.6 % (0,25) | 0.50 ± 1.1 % (0,07) | 0.97 ± 0.4 % (0,06) | 1.00 ± 0.4 % (-0,09) | | 1.06 ± 0.3 % (<0,01) | 1.06 ± 0.4 % (-0,18) | | | 1.10 ± 0.7 % (0,43) | 1.41 ± 1.3 % (1,30) | |  |
|  | QR40 Safire 0 (L-S) | 0.20 ± 12.7 % (0,22) | 0.50 ± 5.3 % (0,24) | 0.98 ± 3.3 % (-0,02) | 1.00 ± 3.0 % (-0,04) | | 1.06 ± 3.0 % (0,02) | 1.06 ± 3.1 % (0,07) | | | 1.09 ± 2.2 % (-0,06) | 1.40 ± 2.8 % (-0,03) | |  |
|  | QR40 Safire 3 (L-S) | 0.20 ± 9.3 % (0,15) | 0.50 ± 3.6 % (0,11) | 0.98 ± 2.1 % (-0,02) | 1.00 ± 2.0 % (-0,04) | | 1.06 ± 2.0 % (-0,03) | 1.06 ± 2.1 % (<0,01) | | | 1.09 ± 1.5 % (-0,05) | 1.40 ± 2.0 % (0,02) | |  |
|  | QR40 Safire 5 (L-S) | 0.20 ± 7.2 % (0,03) | 0.50 ± 2.5 % (-0,12) | 0.98 ± 1.4 % (0,01) | 1.00 ± 1.3 % (-0,02) | | 1.06 ± 1.3 % (-0,05) | 1.06 ± 1.4 % (-0,07) | | | 1.09 ± 1.0 % (-0,04) | 1.40 ± 1.5 % (0,12) | |  |

Table S8. Mean SPR and 87% confidence intervals (± 1.5 SD) and skewness (within parenthesis) measured in SPR images generated with N&A [13], L-S [15,16], and DirectSPR [17] from image data acquired at 40 mGy CTDI_vol_ with different levels of iterative noise reduction (ASiR-V / Safire). Abbreviations: SPR = stopping power ratios; SD = standard deviation; N&A = Näsmark & Andersson (2021); L-S = Landry-Saito; CTDI_vol_ = computed tomography dose index.

| Scanner 1 | **Kernel and noise reduction** | **Lung (inhale)** | | **Lung (exhale)** | | **Adipose** | | **Breast** | | **Liver** | **Muscle** | **Bone 200** | **Bone 800** |
| --- | --- | --- | --- | --- | --- | --- | --- | --- | --- | --- | --- | --- | --- |
|  | Standard ASiR-V 0 (N&A) | 0.21 ± 11.9 % (1,91) | 0.51 ± 3.2 % (1,53) | | 0.97 ± 1.0 % (0,41) | | 1.00 ± 0.8 % (0,09) | | 1.06 ± 0.8 % (0,18) | | 1.06 ± 1.0 % (0,30) | 1.10 ± 3.6 % (-1,22) | 1.4 ± 2.6 % (0,66) |
|  | Standard ASiR-V 40 (N&A) | 0.21 ± 11.9 % (1,95) | 0.51 ± 2.9 % (2,10) | | 0.97 ± 0.9 % (0,48) | | 1.00 ± 0.7 % (0,15) | | 1.06 ± 0.7 % (0,24) | | 1.06 ± 0.8 % (0,45) | 1.10 ± 3.5 % (-1,24) | 1.4 ± 2.6 % (0,67) |
|  | Standard ASiR-V 80 (N&A) | 0.21 ± 11.9 % (1,99) | 0.51 ± 2.6 % (3,19) | | 0.97 ± 0.8 % (0,56) | | 1.00 ± 0.6 % (0,20) | | 1.06 ± 0.6 % (0,29) | | 1.06 ± 0.7 % (0,55) | 1.10 ± 3.5 % (-1,26) | 1.4 ± 2.6 % (0,68) |
|  |  |  |  | |  | |  | |  | |  |  |  |
| Scanner 2 | QR40 Safire 0 (N&A) | 0.20 ± 5.4 % (-0,08) | 0.50 ± 1.6 % (0,01) | | 0.97 ± 0.6 % (0,07) | | 1.00 ± 0.6 % (-0,09) | | 1.06 ± 0.5 % (0,03) | | 1.06 ± 0.6 % (-0,03) | 1.10 ± 0.9 % (0,16) | 1.41 ± 1.5 % (0,76) |
|  | QR40 Safire 3 (N&A) | 0.20 ± 4.7 % (-0,14) | 0.50 ± 1.2 % (0,11) | | 0.97 ± 0.4 % (0,10) | | 1.00 ± 0.4 % (-0,11) | | 1.06 ± 0.4 % (0,04) | | 1.06 ± 0.4 % (-0,12) | 1.10 ± 0.8 % (0,36) | 1.41 ± 1.3 % (1,03) |
|  | QR40 Safire 5 (N&A) | 0.20 ± 4.7 % (0,24) | 0.50 ± 1.1 % (0,28) | | 0.97 ± 0.3 % (0,12) | | 1.00 ± 0.3 % (-0,08) | | 1.06 ± 0.3 % (-0,03) | | 1.06 ± 0.4 % (-0,14) | 1.10 ± 0.7 % (0,48) | 1.41 ± 1.3 % (1,22) |
|  | QR40 Safire 0 (L-S) | 0.19 ± 11.5 % (0,19) | 0.50 ± 4.7 % (0,20) | | 0.98 ± 2.8 % (-0,02) | | 1.00 ± 2.6 % (-0,03) | | 1.06 ± 2.6 % (0,01) | | 1.06 ± 2.7 % (0,04) | 1.09 ± 1.9 % (-0,06) | 1.40 ± 2.5 % (0,01) |
|  | QR40 Safire 3 (L-S) | 0.19 ± 8.6 % (0,12) | 0.50 ± 3.2 % (0,05) | | 0.98 ± 1.9 % (-0,02) | | 1.00 ± 1.7 % (-0,03) | | 1.06 ± 1.7 % (-0,03) | | 1.06 ± 1.8 % (-0,03) | 1.09 ± 1.3 % (-0,05) | 1.40 ± 1.8 % (0,08) |
|  | QR40 Safire 5 (L-S) | 0.19 ± 6.8 % (<0,01) | 0.50 ± 2.2 % (-0,24) | | 0.98 ± 1.2 % (0,03) | | 1.00 ± 1.2 % (-0,01) | | 1.06 ± 1.1 % (-0,04) | | 1.06 ± 1.2 % (-0,05) | 1.09 ± 0.9 % (-0,08) | 1.40 ± 1.3 % (0,12) |
|  | QR40 Safire 0 (DirectSPR) | 0.20 ± 6.5 % (-0,23) | 0.51 ± 1.7 % (-0,06) | | 0.97 ± 1.2 % (-0,25) | | 0.99 ± 1.1 % (-0,05) | | 1.05 ± 0.9 % (-0,06) | | 1.05 ± 1.0 % (-0,18) | 1.09 ± 0.9 % (-0,28) | 1.40 ± 1.1 % (-0,17) |
|  | QR40 Safire 3 (DirectSPR) | 0.20 ± 5.4 % (-0,34) | 0.51 ± 1.2 % (-0,12) | | 0.97 ± 0.9 % (-0,28) | | 0.99 ± 0.8 % (0,03) | | 1.05 ± 0.7 % (-0,01) | | 1.05 ± 0.7 % (-0,25) | 1.09 ± 0.7 % (-0,09) | 1.41 ± 1.0 % (-0,4) |
|  | QR40 Safire 5 (DirectSPR) | 0.20 ± 4.9 % (-0,41) | 0.51 ± 0.9 % (-0,26) | | 0.97 ± 0.7 % (-0,59) | | 0.99 ± 0.7 % (-0,10) | | 1.05 ± 0.6 % (-0,09) | | 1.05 ± 0.6 % (-0,30) | 1.09 ± 0.6 % (-0,18) | 1.40 ± 1.7 % (-1,95) |

Table S9. Mean SPR and 87% confidence intervals (± 1.5 SD) and skewness (within parenthesis) measured in SPR images generated with N&A [13] and L-S [15,16] from image data acquired at 50 mGy CTDI_vol_ with different levels of iterative noise reduction (ASiR-V / Safire). Abbreviations: SPR = stopping power ratios; SD = standard deviation; N&A = Näsmark & Andersson (2021); L-S = Landry-Saito; CTDI_vol_ = computed tomography dose index.

| Scanner 1 | **Kernel and noise reduction** | **Lung (inhale)** | **Lung (exhale)** | | **Adipose** | | **Breast** | | **Liver** | | **Muscle** | | **Bone 200** | **Bone 800** |
| --- | --- | --- | --- | --- | --- | --- | --- | --- | --- | --- | --- | --- | --- | --- |
|  | Standard ASiR-V 0 (N&A) | 0.21 ± 11.8 % (1,95) | 0.51 ± 3.1 % (1,93) | 0.97 ± 1.0 % (0,50) | | 1.00 ± 0.8 % (0,13) | | 1.06 ± 0.7 % (0,23) | | 1.06 ± 0.9 % (0,44) | | 1.10 ± 3.5 % (-1,31) | | 1.41 ± 2.7 % (0,48) |
|  | Standard ASiR-V 40 (N&A) | 0.21 ± 11.8 % (1,99) | 0.51 ± 2.8 % (3,01) | 0.97 ± 0.9 % (0,61) | | 1.00 ± 0.7 % (0,18) | | 1.06 ± 0.7 % (0,29) | | 1.06 ± 0.8 % (0,57) | | 1.10 ± 3.5 % (-1,34) | | 1.41 ± 2.7 % (0,49) |
|  | Standard ASiR-V 80 (N&A) | 0.21 ± 11.8 % (2,02) | 0.51 ± 2.6 % (4,04) | 0.97 ± 0.8 % (0,67) | | 1.00 ± 0.6 % (0,24) | | 1.06 ± 0.6 % (0,35) | | 1.06 ± 0.8 % (0,67) | | 1.10 ± 3.4 % (-1,36) | | 1.41 ± 2.7 % (0,49) |
|  |  |  |  |  | |  | |  | |  | |  | |  |
|  | QR40 Safire 0 (N&A) | 0.20 ± 5.2 % (-0,07) | 0.50 ± 1.5 % (0,02) | 0.97 ± 0.5 % (0,07) | | 1.00 ± 0.5 % (-0,09) | | 1.06 ± 0.5 % (0,05) | | 1.06 ± 0.5 % (-0,04) | | 1.10 ± 0.9 % (0,21) | | 1.40 ± 1.40 % (0,64) |
| Scanner 2 | QR40 Safire 3 (N&A) | 0.20 ± 4.7 % (0,07) | 0.50 ± 1.2 % (0,06) | 0.97 ± 0.4 % (0,03) | | 1.00 ± 0.4 % (-0,12) | | 1.06 ± 0.4 % (0,02) | | 1.06 ± 0.4 % (-0,12) | | 1.10 ± 0.8 % (0,41) | | 1.40 ± 1.3 % (0,86) |
|  | QR40 Safire 5 (N&A) | 0.20 ± 4.4 % (0,04) | 0.50 ± 1.0 % (0,17) | 0.97 ± 0.3 % (0,07) | | 1.00 ± 0.3 % (-0,08) | | 1.06 ± 0.3 % (-0,04) | | 1.06 ± 0.3 % (-0,16) | | 1.10 ± 0.7 % (0,55) | | 1.40 ± 1.2 % (0,8) |
|  | QR40 Safire 0 (L-S) | 0.20 ± 10.8 % (0,17) | 0.50 ± 4.3 % (0,17) | 0.98 ± 2.6 % (-0,01) | | 1.00 ± 2.4 % (-0,04) | | 1.06 ± 2.3 % (-0,01) | | 1.06 ± 2.5 % (0,02) | | 1.09 ± 1.8 % (-0,04) | | 1.40 ± 2.3 % (0,02) |
|  | QR40 Safire 3 (L-S) | 0.19 ± 8.0 % (0,08) | 0.50 ± 2.9 % (0,01) | 0.98 ± 1.7 % (-0,01) | | 1.00 ± 1.6 % (-0,04) | | 1.06 ± 1.5 % (-0,03) | | 1.06 ± 1.6 % (-0,03) | | 1.09 ± 1.2 % (-0,05) | | 1.40 ± 1.7 % (0,08) |
|  | QR40 Safire 5 (L-S) | 0.20 ± 6.5 % (-0,04) | 0.50 ± 2.0 % (-0,30) | 0.98 ± 1.1 % (0,04) | | 1.00 ± 1.1 % (-0,03) | | 1.06 ± 1.1 % (-0,05) | | 1.06 ± 1.1 % (-0,03) | | 1.09 ± 0.8 % (-0,06) | | 1.40 ± 1.3 % (0,14) |

Table S10. Mean SPR and 87% confidence intervals (± 1.5 SD) and skewness (within parenthesis) measured in SPR images generated with N&A [13] and L-S [15,16] from image data acquired at 5 mGy CTDI_vol_ with different levels of iterative noise reduction (ASiR-V / Safire) or deep-learning based noise reduction (TF). Abbreviations: SPR = stopping power ratios; SD = standard deviation; N&A = Näsmark & Andersson (2021); L-S = Landry-Saito; CTDI_vol_ = computed tomography dose index; TF = True Fidelity.

|  | **Kernel and noise reduction** | **Lung (inhale)** | **Lung (exhale)** | **Adipose** | **Breast** | **Liver** | **Muscle** | **Bone 200** | **Bone 800** |
| --- | --- | --- | --- | --- | --- | --- | --- | --- | --- |
| Scanner 1 | Standard ASiR-V 0 (N&A) | 0.21 ± 17.6 % (-0,01) | 0.51 ± 6.7 % (-0,34) | 0.96 ± 2.1 % (0,24) | 0.99 ± 2.1 % (0,17) | 1.05 ± 2.2 % (0,02) | 1.05 ± 2.2 % (0,03) | 1.11 ± 3.0 % (-0,24) | 1.44 ± 2.3 % (0,04) |
|  | Standard ASiR-V 40 (N&A) | 0.21 ± 16.7 % (<0,01) | 0.51 ± 6.9 % (-0,27) | 0.96 ± 1.6 % (0,14) | 0.99 ± 1.6 % (0,09) | 1.05 ± 1.7 % (<0,01) | 1.05 ± 1.7 % (0,01) | 1.11 ± 3.0 % (-0,24) | 1.44 ± 2.3 % (-0,03) |
|  | Standard ASiR-V 80 (N&A) | 0.21 ± 13.1 % (0,10) | 0.51 ± 5.0 % (-0,29) | 0.96 ± 1.4 % (<0,01) | 0.99 ± 1.4 % (0,02) | 1.05 ± 1.4 % (-0,03) | 1.05 ± 1.3 % (0,01) | 1.11 ± 2.3 % (-1,59) | 1.44 ± 1.8 % (-0,23) |
|  | Standard TF low (N&A) | 0.21 ± 18.7 % (0,04) | 0.51 ± 6.7 % (-0,28) | 0.96 ± 1.7 % (0,28) | 0.99 ± 1.6 % (0,22) | 1.05 ± 1.6 % (-0,01) | 1.05 ± 1.7 % (<0,01) | 1.11 ± 2.9 % (-0,26) | 1.44 ± 2.2 % (0,21) |
|  | Standard TF medium (N&A) | 0.21 ± 18.2 % (0,02) | 0.51 ± 6.3 % (-0,41) | 0.96 ± 1.5 % (0,31) | 0.99 ± 1.4 % (0,24) | 1.05 ± 1.5 % (-0,02) | 1.05 ± 1.5 % (-0,01) | 1.11 ± 2.8 % (-0,75) | 1.44 ± 2.1 % (0,45) |
|  | Standard TF high (N&A) | 0.21 ± 17.1 % (0,05) | 0.51 ± 5.8 % (-0,15) | 0.96 ± 1.4 % (0,33) | 0.99 ± 1.3 % (0,30) | 1.05 ± 1.3 % (<0,01) | 1.05 ± 1.3 % (-0,03) | 1.11 ± 2.5 % (-1,17) | 1.44 ± 1.9 % (-0,22) |
|  |  |  |  |  |  |  |  |  |  |
| Scanner 2 | QR40 Safire 0 (N&A) | 0.21 ± 18.8 % (-0,03) | 0.51 ± 8.1 % (0,03) | 0.97 ± 2.9 % (0,12) | 1.00 ± 2.6 % (0,2) | 1.06 ± 2.8 % (0,48) | 1.06 ± 3.1 % (0,61) | 1.10 ± 4.9 % (0,17) | 1.41 ± 4.9 % (0,05) |
|  | QR40 Safire 3 (N&A) | 0.21 ± 13.0 % (0,01) | 0.51 ± 5.7 % (0,05) | 0.97 ± 2.1 % (0,01) | 1.00 ± 1.9 % (0,08) | 1.06 ± 1.9 % (0,06) | 1.06 ± 2.1 % (0,11) | 1.09 ± 3.4 % (0,09) | 1.41 ± 3.7 % (0,08) |
|  | QR40 Safire 5 (N&A) | 0.21 ± 9.8 % (0,04) | 0.51 ± 4.2 % (0,05) | 0.97 ± 1.6 % (-0,10) | 1.00 ± 1.4 % (0,03) | 1.06 ± 1.4 % (0,01) | 1.06 ± 1.5 % (-0,02) | 1.09 ± 2.4 % (0,05) | 1.40 ± 3.0 % (0,15) |
|  | QR40 Safire 0 (L-S) | 0.23 ± 260.0 % (9,19) | 0.51 ± 27.8 % (-0,01) | 0.99 ± 15.8 % (<0,01) | 1.02 ± 14.4 % (<0,01) | 1.09 ± 14.0 % (<0,01) | 1.08 ± 14.8 % (<0,01) | 1.13 ± 13.5 % (0,01) | 1.50 ± 12.3 % (<0,01) |
|  | QR40 Safire 3 (L-S) | 0.19 ± 85.7 % (24,54) | 0.51 ± 18.6 % (<0,01) | 0.99 ± 10.7 % (<0,01) | 1.02 ± 9.6 % (<0,01) | 1.08 ± 9.3 % (<0,01) | 1.08 ± 9.9 % (<0,01) | 1.13 ± 9.1 % (0,01) | 1.50 ± 8.7 % (0,02) |
|  | QR40 Safire 5 (L-S) | 0.19 ± 34.1 % (6,9) | 0.51 ± 12.7 % (0,01) | 0.99 ± 7.3 % (-0,02) | 1.02 ± 6.5 % (-0,01) | 1.08 ± 6.4 % (0,01) | 1.08 ± 6.8 % (<0,01) | 1.13 ± 6.3 % (<0,01) | 1.50 ± 6.4 % (0,02) |

Table 11. Mean SPR and 87% confidence intervals (± 1.5 SD) and skewness (within parenthesis) measured in SPR images generated with N&A [13], L-S [15,16] and DirectSPR [17] from image data acquired at 10 mGy CTDI_vol_ with different levels of iterative noise reduction (ASiR-V / Safire) or deep-learning based noise reduction (TF). Abbreviations: SPR = stopping power ratios; SD = standard deviation; N&A = Näsmark & Andersson (2021); L-S = Landry-Saito; CTDI_vol_ = computed tomography dose index; TF = True Fidelity.

|  | **Kernel and noise reduction** | **Lung (inhale)** | **Lung (exhale)** | **Adipose** | **Breast** | **Liver** | **Muscle** | **Bone 200** | **Bone 800** |
| --- | --- | --- | --- | --- | --- | --- | --- | --- | --- |
| Scanner 1 | Standard ASiR-V 0 (N&A) | 0.21 ± 15.8 % (0,15) | 0.51 ± 6.8 % (-0,19) | 0.96 ± 1.8 % (0,19) | 0.99 ± 1.8 % (0,13) | 1.06 ± 1.8 % (0,01) | 1.05 ± 1.9 % (0,01) | 1.11 ± 3.2 % (-0,33) | 1.43 ± 2.6 % (-0,24) |
|  | Standard ASiR-V 40 (N&A) | 0.21 ± 14.5 % (0,11) | 0.51 ± 6.0 % (0,01) | 0.96 ± 1.4 % (0,09) | 0.99 ± 1.3 % (0,06) | 1.06 ± 1.4 % (<0,01) | 1.05 ± 1.4 % (<0,01) | 1.11 ± 2.9 % (-1,15) | 1.43 ± 2.6 % (-0,43) |
|  | Standard ASiR-V 80 (N&A) | 0.21 ± 11.5 % (0,88) | 0.51 ± 4.3 % (0,37) | 0.96 ± 1.4 % (0,07) | 0.99 ± 1.3 % (<0,01) | 1.06 ± 1.2 % (0,01) | 1.05 ± 1.3 % (<0,01) | 1.11 ± 2.5 % (-2,10) | 1.43 ± 2.3 % (-0,95) |
|  | Standard TF low (N&A) | 0.21 ± 16.2 % (0,09) | 0.51 ± 5.8 % (-0,07) | 0.96 ± 1.4 % (0,17) | 0.99 ± 1.3 % (0,08) | 1.06 ± 1.4 % (-0,02) | 1.05 ± 1.4 % (-0,01) | 1.11 ± 3.0 % (-0,72) | 1.43 ± 2.6 % (-0,27) |
|  | Standard TF medium (N&A) | 0.21 ± 15.3 % (0,15) | 0.51 ± 5.2 % (0,09) | 0.96 ± 1.3 % (0,19) | 0.99 ± 1.3 % (0,06) | 1.06 ± 1.3 % (-0,06) | 1.05 ± 1.3 % (-0,02) | 1.11 ± 2.8 % (-1,39) | 1.43 ± 2.4 % (-0,58) |
|  | Standard TF high (N&A) | 0.21 ± 14.2 % (0,35) | 0.51 ± 4.8 % (0,15) | 0.96 ± 1.3 % (0,16) | 0.99 ± 1.3 % (0,10) | 1.06 ± 1.3 % (-0,01) | 1.05 ± 1.2 % (-0,02) | 1.11 ± 2.6 % (-1,84) | 1.43 ± 2.3 % (-0,86) |
|  |  |  |  |  |  |  |  |  |  |
| Scanner 2 | QR40 Safire 0 (N&A) | 0.21 ± 13.3 % (<0,01) | 0.51 ± 5.8 % (0,05) | 0.97 ± 2.2 % (-0,02) | 1.00 ± 1.9 % (0,12) | 1.06 ± 2.0 % (0,07) | 1.06 ± 2.1 % (0,11) | 1.09 ± 3.5 % (0,11) | 1.40 ± 3.7 % (0,08) |
|  | QR40 Safire 3 (N&A) | 0.21 ± 9.4 % (0,04) | 0.51 ± 4.1 % (0,05) | 0.97 ± 1.6 % (-0,08) | 1.00 ± 1.4 % (0,04) | 1.06 ± 1.4 % (<0,01) | 1.06 ± 1.5 % (-0,03) | 1.09 ± 2.4 % (0,04) | 1.40 ± 2.8 % (0,13) |
|  | QR40 Safire 5 (N&A) | 0.21 ± 7.4 % (0,09) | 0.51 ± 3.1 % (0,09) | 0.97 ± 1.2 % (-0,13) | 1.00 ± 1.0 % (-0,02) | 1.06 ± 1.1 % (<0,01) | 1.06 ± 1.1 % (-0,08) | 1.09 ± 1.7 % (0,07) | 1.40 ± 2.3 % (0,26) |
|  | QR40 Safire 0 (L-S) | 0.19 ± 103.2 % (22,64) | 0.51 ± 19.7 % (<0,01) | 0.99 ± 11.3 % (-0,01) | 1.02 ± 10.2 % (-0,01) | 1.08 ± 10.0 % (<0,01) | 1.08 ± 10.5 % (<0,01) | 1.13 ± 9.6 % (0,01) | 1.50 ± 8.9 % (0,01) |
|  | QR40 Safire 3 (L-S) | 0.19 ± 33.3 % (1,77) | 0.51 ± 13.2 % (<0,01) | 0.99 ± 7.5 % (<0,01) | 1.02 ± 6.8 % (-0,01) | 1.08 ± 6.6 % (0,01) | 1.08 ± 7.0 % (<0,01) | 1.13 ± 6.4 % (0,01) | 1.50 ± 6.2 % (0,01) |
|  | QR40 Safire 5 (L-S) | 0.19 ± 22.4 % (<0,01) | 0.51 ± 9.0 % (0,01) | 0.99 ± 5.1 % (-0,01) | 1.02 ± 4.5 % (0,02) | 1.08 ± 4.5 % (0,01) | 1.08 ± 4.8 % (0,01) | 1.13 ± 4.4 % (-0,01) | 1.50 ± 4.5 % (0,01) |
|  | QR40 Safire 0 (DirectSPR) | 0.20 ± 16.0 % (-0,02) | 0.51 ± 6.6 % (0,01) | 0.97 ± 4.5 % (-0,05) | 1.00 ± 4.2 % (0,03) | 1.05 ± 4.0 % (0,24) | 1.05 ± 4.1 % (0,23) | 1.08 ± 3.1 % (<0,01) | 1.38 ± 3.0 % (-0,01) |
|  | QR40 Safire 3 (DirectSPR) | 0.20 ± 11.2 % (-0,02) | 0.51 ± 4.5 % (<0,01) | 0.97 ± 3.4 % (-0,05) | 0.99 ± 3.1 % (0,07) | 1.05 ± 2.8 % (0,29) | 1.04 ± 2.9 % (0,28) | 1.08 ± 2.2 % (-0,02) | 1.38 ± 2.3 % (-0,01) |
|  | QR40 Safire 5 (DirectSPR) | 0.20 ± 7.9 % (-0,06) | 0.51 ± 3.0 % (0,01) | 0.97 ± 2.5 % (-0,05) | 1.00 ± 2.3 % (0,08) | 1.05 ± 2.0 % (0,32) | 1.04 ± 2.0 % (0,26) | 1.08 ± 1.6 % (-0,02) | 1.38 ± 1.8 % (-0,06) |

Table 12. Mean SPR and 87% confidence intervals (± 1.5 SD) and skewness (within parenthesis) measured in SPR images generated with N&A [13] and L-S [15,16] from image data acquired with 15 mGy CTDI_vol_ with different levels of iterative noise reduction (ASiR-V / Safire) or deep-learning based noise reduction (TF). Abbreviations: SPR = stopping power ratios; SD = standard deviation; N&A = Näsmark & Andersson (2021); L-S = Landry-Saito; CTDI_vol_ = computed tomography dose index; TF = True Fidelity.

|  | **Kernel and noise reduction** | **Lung (inhale)** | **Lung (exhale)** | **Adipose** | **Breast** | **Liver** | **Muscle** | **Bone 200** | **Bone 800** |
| --- | --- | --- | --- | --- | --- | --- | --- | --- | --- |
| Scanner 1 | Standard ASiR-V 0 (N&A) | 0.21 ± 15.7 % (0,05) | 0.51 ± 6.7 % (-0,21) | 0.97 ± 1.6 % (0,18) | 0.99 ± 1.5 % (0,1) | 1.06 ± 1.6 % (0,03) | 1.06 ± 1.7 % (0,03) | 1.11 ± 3.0 % (-0,25) | 1.43 ± 2.4 % (-0,14) |
|  | Standard ASiR-V 40 (N&A) | 0.21 ± 13.5 % (0,35) | 0.51 ± 5.4 % (0,46) | 0.97 ± 1.2 % (0,09) | 0.99 ± 1.2 % (0,05) | 1.06 ± 1.3 % (<0,01) | 1.06 ± 1.3 % (0,01) | 1.11 ± 2.5 % (-1,38) | 1.43 ± 2.3 % (-0,56) |
|  | Standard ASiR-V 80 (N&A) | 0.21 ± 11.1 % (1,32) | 0.51 ± 4.0 % (1,53) | 0.97 ± 1.3 % (0,05) | 0.99 ± 1.1 % (-0,04) | 1.06 ± 1.0 % (<0,01) | 1.06 ± 1.2 % (0,02) | 1.11 ± 2.1 % (-2,56) | 1.43 ± 2.0 % (-1,18) |
|  | Standard TF low (N&A) | 0.21 ± 15.3 % (0,08) | 0.51 ± 5.4 % (0,29) | 0.97 ± 1.3 % (0,15) | 0.99 ± 1.2 % (0,05) | 1.06 ± 1.3 % (-0,04) | 1.06 ± 1.4 % (-0,03) | 1.11 ± 2.5 % (-1,05) | 1.43 ± 2.3 % (-0,40) |
|  | Standard TF medium (N&A) | 0.21 ± 14.3 % (0,26) | 0.51 ± 4.8 % (0,72) | 0.97 ± 1.2 % (0,13) | 0.99 ± 1.2 % (0,07) | 1.06 ± 1.3 % (-0,03) | 1.06 ± 1.3 % (-0,02) | 1.11 ± 2.4 % (-1,65) | 1.43 ± 2.2 % (-0,52) |
|  | Standard TF high (N&A) | 0.21 ± 13.9 % (0,46) | 0.51 ± 4.5 % (1,09) | 0.97 ± 1.3 % (0,13) | 0.99 ± 1.3 % (0,05) | 1.06 ± 1.3 % (-0,03) | 1.06 ± 1.2 % (-0,03) | 1.11 ± 2.2 % (-1,96) | 1.43 ± 2.1 % (-0,90) |
|  |  |  |  |  |  |  |  |  |  |
| Scanner 2 | QR40 Safire 0 (N&A) | 0.21 ± 11.1 % (0,02) | 0.51 ± 4.8 % (0,06) | 0.97 ± 1.8 % (-0,07) | 1.00 ± 1.6 % (0,07) | 1.06 ± 1.6 % (0,03) | 1.06 ± 1.7 % (-0,01) | 1.09 ± 2.8 % (0,05) | 1.40 ± 3.1 % (0,12) |
|  | QR40 Safire 3 (N&A) | 0.21 ± 8.1 % (0,08) | 0.51 ± 3.4 % (0,07) | 0.97 ± 1.3 % (-0,20) | 1.00 ± 1.1 % (0,02) | 1.06 ± 1.2 % (0,01) | 1.06 ± 1.2 % (-0,10) | 1.09 ± 2.0 % (<0,01) | 1.40 ± 2.5 % (0,21) |
|  | QR40 Safire 5 (N&A) | 0.21 ± 6.5 % (0,13) | 0.51 ± 2.7 % (0,15) | 0.97 ± 1.0 % (-0,27) | 1.00 ± 0.9 % (-0,03) | 1.06 ± 0.9 % (0,05) | 1.06 ± 0.9 % (-0,15) | 1.09 ± 1.4 % (0,13) | 1.40 ± 2.1 % (0,39) |
|  | QR40 Safire 0 (L-S) | 0.19 ± 49.5 % (17,52) | 0.51 ± 16.1 % (-0,01) | 0.99 ± 9.2 % (-0,01) | 1.02 ± 8.3 % (<0,01) | 1.08 ± 8.1 % (-0,01) | 1.08 ± 8.6 % (0,01) | 1.13 ± 7.8 % (<0,01) | 1.49 ± 7.2 % (0,01) |
|  | QR40 Safire 3 (L-S) | 0.19 ± 27.1 % (<0,01) | 0.51 ± 10.8 % (<0,01) | 0.99 ± 6.1 % (<0,01) | 1.02 ± 5.5 % (<0,01) | 1.08 ± 5.4 % (0,02) | 1.08 ± 5.7 % (0,01) | 1.13 ± 5.3 % (<0,01) | 1.49 ± 5.1 % (<0,01) |
|  | QR40 Safire 5 (L-S) | 0.19 ± 18.5 % (<0,01) | 0.51 ± 7.3 % (<0,01) | 0.99 ± 4.2 % (<0,01) | 1.02 ± 3.7 % (-0,02) | 1.08 ± 3.6 % (<0,01) | 1.08 ± 3.9 % (-0,01) | 1.13 ± 3.6 % (<0,01) | 1.49 ± 3.7 % (0,01) |

**Section S6. Range tables**

Tables S13-S18 shows the mean SPR with 87% confidence intervals (± 1.5 SD) and skewness (within parenthesis) measured in the SPR images generated with N&A [13], L-S [15,16] and DirectSPR [17]. The SPR images were generated from image data acquired with 30, 40, 50 mGy CTDI_vol_ for the head phantom and 5, 10, and 15 mGy CTDI_vol_ for the Body phantom, with different levels of iterative or deep-learning based noise reduction. To account for intra- and inter-scan variabilities, the mean values and confidence intervals have been averaged over 30 identical acquisitions.

Table S13. Mean range with 87% confidence interval (± 1.5 SD) and skewness (within parenthesis) for protons passing through tissue equivalent phantom inserts in simulations based on SPR maps generated from image data acquired with 30 mGy CTDI_vol._ SPR maps were generated with N&A [13] or L-S [15,16], with different levels of iterative noise reduction (ASiR-V / Safire). Abbreviations: SD = standard deviation; SPR = stopping power ratios; CTDI_vol_ = computed tomography dose index; N&A = Näsmark & Andersson (2021); L-S = Landry-Saito.

| Scanner 1 | **Kernel and noise reduction** | **Lung (inhale)** | **Lung (exhale)** | **Adipose** | **Breast** | **Liver** | **Muscle** | **Bone 200** | **Bone 800** |
| --- | --- | --- | --- | --- | --- | --- | --- | --- | --- |
|  | Standard ASiR-V 0 (N&A) | 12.2 cm ± 0.4 % (0,29) | 10.9 cm ± 0.5 % (0,46) | 8.9 cm ± 0.3 % (0,44) | 8.8 cm ± 0.6 % (0,28) | 8.5 cm ± 0.5 % (0,24) | 8.4 cm ± 0.2 % (0,05) | 8.4 cm ± 0.4 % (0,14) | 7.4 cm ± 0.2 % (0,56) |
|  | Standard ASiR-V 40 (N&A) | 12.2 cm ± 0.4 % (0,29) | 10.9 cm ± 0.5 % (0,39) | 8.9 cm ± 0.3 % (0,49) | 8.8 cm ± 0.6 % (0,39) | 8.5 cm ± 0.5 % (0,21) | 8.4 cm ± 0.2 % (0,18) | 8.4 cm ± 0.4 % (0,04) | 7.4 cm ± 0.2 % (0,46) |
|  | Standard ASiR-V 80 (N&A) | 12.2 cm ± 0.4 % (0,24) | 10.9 cm ± 0.5 % (0,30) | 8.9 cm ± 0.3 % (0,63) | 8.8 cm ± 0.6 % (0,13) | 8.5 cm ± 0.5 % (0,29) | 8.4 cm ± 0.2 % (0,17) | 8.4 cm ± 0.4 % (0,04) | 7.4 cm ± 0.2 % (0,46) |
|  |  |  |  |  |  |  |  |  |  |
| Scanner 2 | QR40 Safire 0 (N&A) | 12.0 cm ± 0.7 % (0,22) | 10.6 cm ± 0.7 % (0,69) | 8.5 cm ± 0.6 % (0,35) | 8.4 cm ± 0.8 % (0,22) | 8.2 cm ± 1.6 % (0,76) | 8.3 cm ± 0.7 % (0,20) | 8.1 cm ± 1.5 % (0,29) | 7.4 cm ± 0.9 % (0,17) |
|  | QR40 Safire 3 (N&A) | 12.0 cm ± 0.7 % (0,37) | 10.6 cm ± 0.8 % (0,63) | 8.5 cm ± 0.6 % (0,45) | 8.4 cm ± 0.7 % (0,21) | 8.2 cm ± 1.4 % (0,51) | 8.3 cm ± 0.7 % (0,25) | 8.1 cm ± 1.3 % (0,26) | 7.4 cm ± 0.9 % (0,47) |
|  | QR40 Safire 5 (N&A) | 12.1 cm ± 0.8 % (0,48) | 10.7 cm ± 0.8 % (0,62) | 8.5 cm ± 0.7 % (0,31) | 8.4 cm ± 0.7 % (0,21) | 8.2 cm ± 1.2 % (0,50) | 8.3 cm ± 0.6 % (0,27) | 8.1 cm ± 1.2 % (0,15) | 7.4 cm ± 0.9 % (0,35) |
|  | QR40 Safire 0 (L-S) | 11.9 cm ± 0.9 % (0,29) | 10.5 cm ± 0.8 % (0,99) | 8.3 cm ± 0.8 % (0,60) | 8.3 cm ± 1.0 % (0,28) | 8.0 cm ± 2.1 % (0,98) | 8.2 cm ± 0.7 % (0,61) | 8.1 cm ± 2.1 % (0,46) | 7.3 cm ± 0.9 % (0,35) |
|  | QR40 Safire 3 (L-S) | 11.9 cm ± 0.8 % (0,16) | 10.5 cm ± 0.7 % (0,95) | 8.3 cm ± 0.6 % (0,41) | 8.3 cm ± 0.9 % (0,29) | 8.0 cm ± 1.8 % (0,90) | 8.2 cm ± 0.6 % (0,27) | 8.1 cm ± 1.8 % (0,32) | 7.3 cm ± 0.8 % (0,37) |
|  | QR40 Safire 5 (L-S) | 11.9 cm ± 0.7 % (0,27) | 10.5 cm ± 0.7 % (0,92) | 8.3 cm ± 0.6 % (0,29) | 8.3 cm ± 0.9 % (0,43) | 8.0 cm ± 1.5 % (0,99) | 8.2 cm ± 0.6 % (0,22) | 8.0 cm ± 1.7 % (0,34) | 7.3 cm ± 0.8 % (0,41) |

Table S14. Mean range with 87% confidence interval (± 1.5 SD) and skewness (within parenthesis) for protons passing through tissue equivalent phantom inserts in simulations based on SPR maps generated from image data acquired with 40 mGy CTDI_vol._ SPR maps were generated with N&A [13], L-S [15,16] or DirectSPR [17], with different levels of iterative noise reduction (ASiR-V / Safire). Abbreviations: SD = standard deviation; SPR = stopping power ratios; CTDI_vol_ = computed tomography dose index; N&A = Näsmark & Andersson (2021); L-S = Landry-Saito.

| Scanner 1 | **Kernel and noise reduction** | **Lung (inhale)** | **Lung (exhale)** | **Adipose** | **Breast** | **Liver** | **Muscle** | **Bone 200** | **Bone 800** |
| --- | --- | --- | --- | --- | --- | --- | --- | --- | --- |
|  | Standard ASiR-V 0 (N&A) | 12.2 cm ± 0.3 % (0,47) | 10.9 cm ± 0.4 % (0,41) | 8.9 cm ± 0.3 % (0,34) | 8.8 cm ± 0.6 % (0,34) | 8.5 cm ± 0.4 % (0,44) | 8.5 cm ± 0.2 % (0,31) | 8.4 cm ± 0.3 % (0,05) | 7.5 cm ± 0.3 % (0,47) |
|  | Standard ASiR-V 40 (N&A) | 12.2 cm ± 0.3 % (0,37) | 10.9 cm ± 0.4 % (0,50) | 8.9 cm ± 0.3 % (0,36) | 8.8 cm ± 0.6 % (0,44) | 8.5 cm ± 0.4 % (0,28) | 8.5 cm ± 0.2 % (0,31) | 8.4 cm ± 0.3 % (0,05) | 7.5 cm ± 0.3 % (0,55) |
|  | Standard ASiR-V 80 (N&A) | 12.2 cm ± 0.3 % (0,27) | 10.9 cm ± 0.4 % (0,47) | 8.9 cm ± 0.3 % (0,43) | 8.8 cm ± 0.6 % (0,24) | 8.5 cm ± 0.4 % (0,47) | 8.5 cm ± 0.2 % (0,28) | 8.4 cm ± 0.3 % (0,18) | 7.5 cm ± 0.3 % (0,54) |
|  |  |  |  |  |  |  |  |  |  |
| Scanner 2 | QR40 Safire 0 (N&A) | 12.0 cm ± 0.7 % (0,41) | 10.6 cm ± 0.7 % (0,80) | 8.5 cm ± 0.7 % (0,44) | 8.4 cm ± 0.8 % (0,48) | 8.1 cm ± 1.5 % (0,75) | 8.3 cm ± 0.6 % (0,29) | 8.1 cm ± 1.4 % (0,25) | 7.4 cm ± 0.8 % (0,49) |
|  | QR40 Safire 3 (N&A) | 12.0 cm ± 0.7 % (0,38) | 10.6 cm ± 0.8 % (0,76) | 8.5 cm ± 0.6 % (0,43) | 8.4 cm ± 0.7 % (0,29) | 8.2 cm ± 1.3 % (0,56) | 8.3 cm ± 0.6 % (0,28) | 8.1 cm ± 1.3 % (0,22) | 7.4 cm ± 0.8 % (0,25) |
|  | QR40 Safire 5 (N&A) | 12.1 cm ± 0.7 % (0,22) | 10.6 cm ± 0.8 % (0,92) | 8.5 cm ± 0.6 % (0,23) | 8.4 cm ± 0.8 % (0,32) | 8.2 cm ± 1.4 % (0,61) | 8.3 cm ± 0.6 % (0,17) | 8.1 cm ± 1.2 % (0,37) | 7.4 cm ± 0.9 % (0,26) |
|  | QR40 Safire 0 (L-S) | 11.9 cm ± 0.8 % (0,26) | 10.5 cm ± 0.8 % (0,92) | 8.3 cm ± 0.6 % (0,54) | 8.3 cm ± 1.0 % (0,41) | 8.0 cm ± 2.0 % (0,99) | 8.2 cm ± 0.6 % (0,51) | 8.1 cm ± 2.0 % (0,41) | 7.3 cm ± 0.9 % (0,38) |
|  | QR40 Safire 3 (L-S) | 11.9 cm ± 0.7 % (0,27) | 10.4 cm ± 0.7 % (0,93) | 8.4 cm ± 0.6 % (0,47) | 8.3 cm ± 0.8 % (0,31) | 8.0 cm ± 1.7 % (0,97) | 8.2 cm ± 0.6 % (0,46) | 8.1 cm ± 1.8 % (0,42) | 7.3 cm ± 0.8 % (0,34) |
|  | QR40 Safire 5 (L-S) | 11.9 cm ± 0.7 % (0,26) | 10.4 cm ± 0.7 % (0,91) | 8.4 cm ± 0.6 % (0,42) | 8.3 cm ± 0.8 % (0,41) | 8.0 cm ± 1.7 % (0,91) | 8.2 cm ± 0.5 % (0,38) | 8.1 cm ± 1.8 % (0,43) | 7.3 cm ± 0.8 % (0,29) |
|  | QR40 Safire 0 (DirectSPR) | 12.1 cm ± 0.9 % (0,28) | 10.7 cm ± 0.8 % (0,90) | 8.5 cm ± 0.6 % (0,53) | 8.4 cm ± 0.9 % (0,38) | 8.2 cm ± 1.8 % (0,76) | 8.4 cm ± 0.6 % (0,52) | 8.1 cm ± 1.7 % (0,41) | 7.4 cm ± 0.7 % (0,27) |
|  | QR40 Safire 3 (DirectSPR) | 12.2 cm ± 0.7 % (0,32) | 10.8 cm ± 0.7 % (0,99) | 8.6 cm ± 0.5 % (0,61) | 8.6 cm ± 0.8 % (0,12) | 8.3 cm ± 1.7 % (0,68) | 8.4 cm ± 0.6 % (0,53) | 8.2 cm ± 1.7 % (0,25) | 7.4 cm ± 0.7 % (0,25) |
|  | QR40 Safire 5 (DirectSPR) | 12.3 cm ± 0.7 % (0,53) | 10.9 cm ± 0.6 % (0,66) | 8.6 cm ± 0.6 % (0,77) | 8.6 cm ± 0.8 % (0,21) | 8.4 cm ± 1.6 % (0,75) | 8.4 cm ± 0.5 % (0,50) | 8.3 cm ± 1.5 % (0,31) | 7.4 cm ± 0.7 % (0,41) |

Table S15. Mean range with 87% confidence interval (± 1.5 SD) and skewness (within parenthesis) for protons passing through tissue equivalent phantom inserts in simulations based on SPR maps generated from image data acquired with 50 mGy CTDI_vol._ SPR maps were generated with N&A [13] or L-S [8,9], with different levels of iterative noise reduction (ASiR-V / Safire). Abbreviations: SD = standard deviation; SPR = stopping power ratios; N&A = Näsmark & Andersson (2021); L-S=Landry-Saito; CTDI_vol_ = computed tomography dose index.

| Scanner 1 | **Kernel and noise reduction** | | **Lung (inhale)** | | **Lung (exhale)** | | **Adipose** | | **Breast** | | **Liver** | | **Muscle** | | **Bone 200** | | **Bone 800** |
| --- | --- | --- | --- | --- | --- | --- | --- | --- | --- | --- | --- | --- | --- | --- | --- | --- | --- |
|  | Standard ASiR-V 0 (N&A) | 12.2 cm ± 0.3 % (0,18) | | 10.9 cm ± 0.4 % (0,48) | | 8.9 cm ± 0.3 % (0,67) | | 8.8 cm ± 0.5 % (0,10) | | 8.5 cm ± 0.5 % (0,32) | | 8.5 cm ± 0.2 % (-0,09) | | 8.4 cm ± 0.3 % (0,04) | | 7.5 cm ± 0.4 % (0,65) | |
|  | Standard ASiR-V 40 (N&A) | 12.2 cm ± 0.3 % (0,36) | | 10.9 cm ± 0.4 % (0,51) | | 8.9 cm ± 0.3 % (0,63) | | 8.8 cm ± 0.5 % (0,27) | | 8.5 cm ± 0.5 % (0,25) | | 8.5 cm ± 0.2 % (-0,03) | | 8.4 cm ± 0.3 % (-0,06) | | 7.5 cm ± 0.4 % (0,59) | |
|  | Standard ASiR-V 80 (N&A) | 12.2 cm ± 0.3 % (0,37) | | 10.9 cm ± 0.4 % (0,65) | | 8.9 cm ± 0.3 % (0,64) | | 8.8 cm ± 0.5 % (0,23) | | 8.5 cm ± 0.5 % (0,24) | | 8.5 cm ± 0.2 % (-0,10) | | 8.4 cm ± 0.3 % (-0,01) | | 7.5 cm ± 0.4 % (0,41) | |
|  |  |  | |  | |  | |  | |  | |  | |  | |  | |
| Scanner 2 | QR40 Safire 0 (N&A) | 12.0 cm ± 0.7 % (0,30) | | 10.6 cm ± 0.7 % (0,85) | | 8.5 cm ± 0.6 % (0,35) | | 8.4 cm ± 0.8 % (0,34) | | 8.1 cm ± 1.6 % (0,69) | | 8.3 cm ± 0.6 % (0,27) | | 8.1 cm ± 1.5 % (0,38) | | 7.4 cm ± 0.8 % (0,48) | |
|  | QR40 Safire 3 (N&A) | 12.1 cm ± 0.7 % (0,33) | | 10.7 cm ± 0.9 % (0,98) | | 8.6 cm ± 0.6 % (0,37) | | 8.4 cm ± 0.7 % (0,18) | | 8.2 cm ± 1.6 % (0,66) | | 8.3 cm ± 0.7 % (0,17) | | 8.1 cm ± 1.4 % (0,10) | | 7.5 cm ± 0.8 % (0,34) | |
|  | QR40 Safire 5 (N&A) | 12.1 cm ± 0.8 % (0,34) | | 10.6 cm ± 0.9 % (0,83) | | 8.5 cm ± 0.6 % (0,37) | | 8.4 cm ± 0.7 % (0,26) | | 8.2 cm ± 1.5 % (0,61) | | 8.3 cm ± 0.7 % (0,28) | | 8.1 cm ± 1.3 % (0,30) | | 7.4 cm ± 0.9 % (0,21) | |
|  | QR40 Safire 0 (L-S) | 11.9 cm ± 0.8 % (0,52) | | 10.5 cm ± 0.8 % (0,98) | | 8.3 cm ± 0.6 % (0,38) | | 8.3 cm ± 0.9 % (0,58) | | 8.0 cm ± 2.1 % (0,89) | | 8.2 cm ± 0.6 % (0,59) | | 8.1 cm ± 2.0 % (0,27) | | 7.3 cm ± 0.8 % (0,41) | |
|  | QR40 Safire 3 (L-S) | 12.0 cm ± 0.8 % (0,35) | | 10.5 cm ± 0.7 % (0,97) | | 8.4 cm ± 0.6 % (0,46) | | 8.4 cm ± 0.8 % (0,53) | | 8.1 cm ± 2.1 % (0,89) | | 8.2 cm ± 0.5 % (0,43) | | 8.1 cm ± 2.0 % (0,35) | | 7.3 cm ± 0.7 % (0,33) | |
|  | QR40 Safire 5 (L-S) | 11.9 cm ± 0.7 % (0,20) | | 10.4 cm ± 0.7 % (1,01) | | 8.4 cm ± 0.5 % (0,27) | | 8.3 cm ± 0.8 % (0,47) | | 8.0 cm ± 1.9 % (0,90) | | 8.2 cm ± 0.6 % (0,46) | | 8.1 cm ± 1.8 % (0,47) | | 7.3 cm ± 0.8 % (0,32) | |

Table S16. Mean range with 87% confidence interval (± 1.5 SD) and skewness (within parenthesis) for protons passing through tissue equivalent phantom inserts in simulations based on SPR maps generated from image data acquired with 5 mGy CTDI_vol._ SPR maps were generated with N&A [13] or L-S [8,9], with different levels of iterative noise reduction (ASiR-V / Safire) or deep-learning based noise reduction (TF). Abbreviations: SD = standard deviation; SPR = stopping power ratios. N&A = Näsmark & Andersson (2021); L-S = Landry-Saito; CTDI_vol_ = computed tomography dose index; TF = True Fidelity.

|  | **Kernel and noise reduction Lung (inhale)** | | **Lung (exhale)** | **Adipose** | **Breast** | **Liver** | **Muscle** | **Bone 200** | **Bone 800** |
| --- | --- | --- | --- | --- | --- | --- | --- | --- | --- |
| Scanner 1 | Standard ASiR-V 0 (N&A) | 12.2 cm ± 0.6 % (0,03) | 11.0 cm ± 0.6 % (0,28) | 8.8 cm ± 0.6 % (0,48) | 8.8 cm ± 0.9 % (0,20) | 8.4 cm ± 0.9 % (0,52) | 8.4 cm ± 0.6 % (0,11) | 8.2 cm ± 0.9 % (-0,01) | 7.3 cm ± 0.7 % (0,21) |
|  | Standard ASiR-V 40 (N&A) | 12.2 cm ± 0.6 % (0,08) | 11.0 cm ± 0.7 % (0,22) | 8.8 cm ± 0.6 % (0,59) | 8.7 cm ± 0.8 % (0,23) | 8.4 cm ± 0.9 % (0,43) | 8.4 cm ± 0.5 % (0,14) | 8.2 cm ± 0.9 % (0,16) | 7.3 cm ± 0.6 % (0,19) |
|  | Standard ASiR-V 80 (N&A) | 12.2 cm ± 0.6 % (0,15) | 11.0 cm ± 0.6 % (0,20) | 8.8 cm ± 0.6 % (0,57) | 8.7 cm ± 0.8 % (0,04) | 8.4 cm ± 0.9 % (0,48) | 8.4 cm ± 0.5 % (0,12) | 8.2 cm ± 0.9 % (-0,03) | 7.3 cm ± 0.6 % (0,16) |
|  | Standard TF low (N&A) | 12.2 cm ± 0.6 % (0,14) | 10.9 cm ± 0.6 % (0,16) | 8.8 cm ± 0.6 % (0,46) | 8.7 cm ± 0.8 % (0,35) | 8.4 cm ± 0.9 % (0,56) | 8.4 cm ± 0.5 % (-0,02) | 8.2 cm ± 0.9 % (0,21) | 7.3 cm ± 0.6 % (0,28) |
|  | Standard TF medium (N&A) | 12.2 cm ± 0.6 % (0,10) | 10.9 cm ± 0.7 % (0,31) | 8.8 cm ± 0.6 % (0,61) | 8.7 cm ± 0.9 % (0,25) | 8.4 cm ± 0.9 % (0,43) | 8.4 cm ± 0.5 % (-0,09) | 8.2 cm ± 0.9 % (0,26) | 7.3 cm ± 0.6 % (0,16) |
|  | Standard TF high (N&A) | 12.2 cm ± 0.6 % (-0,02) | 10.9 cm ± 0.6 % (0,26) | 8.8 cm ± 0.6 % (0,53) | 8.7 cm ± 0.9 % (0,38) | 8.4 cm ± 0.9 % (0,48) | 8.4 cm ± 0.5 % (0,12) | 8.2 cm ± 0.9 % (0,07) | 7.3 cm ± 0.6 % (0,11) |
|  |  |  |  |  |  |  |  |  |  |
| Scanner 2 | QR40 Safire 0 (N&A) | 12.1 cm ± 0.8 % (0,35) | 10.8 cm ± 0.7 % (0,15) | 8.7 cm ± 0.9 % (0,32) | 8.6 cm ± 1.1 % (0,40) | 8.4 cm ± 1.2 % (0,45) | 8.3 cm ± 0.8 % (0,19) | 8.2 cm ± 1.5 % (0,43) | 7.5 cm ± 0.8 % (0,38) |
|  | QR40 Safire 3 (N&A) | 12.1 cm ± 0.7 % (0,22) | 10.8 cm ± 0.7 % (0,33) | 8.7 cm ± 0.8 % (0,32) | 8.6 cm ± 0.8 % (0,52) | 8.4 cm ± 1.1 % (0,38) | 8.4 cm ± 0.8 % (0,29) | 8.2 cm ± 1.2 % (0,10) | 7.5 cm ± 0.7 % (0,35) |
|  | QR40 Safire 5 (N&A) | 12.1 cm ± 0.6 % (0,33) | 10.8 cm ± 0.7 % (0,39) | 8.7 cm ± 0.8 % (0,33) | 8.6 cm ± 0.9 % (0,29) | 8.4 cm ± 1.0 % (0,29) | 8.3 cm ± 0.8 % (0,27) | 8.2 cm ± 1.3 % (0,22) | 7.5 cm ± 0.7 % (0,22) |
|  | QR40 Safire 0 (L-S) | 11.8 cm ± 1.4 % (0,38) | 10.6 cm ± 1.1 % (0,19) | 8.4 cm ± 1.3 % (0,34) | 8.4 cm ± 1.6 % (0,42) | 8.2 cm ± 1.7 % (0,48) | 8.1 cm ± 1.2 % (0,16) | 7.9 cm ± 2.0 % (0,32) | 7.1 cm ± 1.1 % (0,50) |
|  | QR40 Safire 3 (L-S) | 12.0 cm ± 1.4 % (0,22) | 10.7 cm ± 1.0 % (0,35) | 8.4 cm ± 1.2 % (0,36) | 8.4 cm ± 1.6 % (0,34) | 8.2 cm ± 1.5 % (0,36) | 8.1 cm ± 1.1 % (0,44) | 8.0 cm ± 2.0 % (0,30) | 7.1 cm ± 1.0 % (0,44) |
|  | QR40 Safire 5 (L-S) | 12.0 cm ± 1.1 % (0,44) | 10.6 cm ± 0.9 % (0,37) | 8.4 cm ± 1.1 % (0,36) | 8.4 cm ± 1.3 % (0,18) | 8.2 cm ± 1.5 % (0,48) | 8.1 cm ± 1.0 % (0,28) | 7.9 cm ± 2.0 % (0,33) | 7.1 cm ± 0.9 % (0,33) |

Table S17. Mean range with 87% confidence interval (± 1.5 SD) and skewness (within parenthesis) for protons passing through tissue equivalent phantom inserts in simulations based on SPR maps generated from image data acquired with 10 mGy CTDI_vol._ SPR maps were generated with N&A [12], L-S [15,16] or DirectSPR [17], with different levels of iterative (ASiR-V / Safire) or deep-learning based noise reduction (TF). Abbreviations: SD = standard deviation; SPR = stopping power ratios; N&A = Näsmark & Andersson (2021); L-S = Landry-Saito; CTDI_vol_ = computed tomography dose index; TF = True Fidelity.

|  | **Kernel and noise reduction Lung (inhale)** | | **Lung (exhale)** | **Adipose** | **Breast** | **Liver** | **Muscle** | **Bone 200** | **Bone 800** |
| --- | --- | --- | --- | --- | --- | --- | --- | --- | --- |
| Scanner 1 | Standard ASiR-V 0 (N&A) | 12.2 cm ± 0.4 % (0,51) | 10.9 cm ± 0.5 % (0,36) | 8.8 cm ± 0.4 % (0,34) | 8.7 cm ± 0.6 % (0,28) | 8.5 cm ± 0.6 % (0,49) | 8.4 cm ± 0.3 % (0,12) | 8.2 cm ± 0.7 % (0,07) | 7.3 cm ± 0.4 % (0,45) |
|  | Standard ASiR-V 40 (N&A) | 12.2 cm ± 0.4 % (0,63) | 10.9 cm ± 0.4 % (0,50) | 8.8 cm ± 0.4 % (0,37) | 8.7 cm ± 0.6 % (0,19) | 8.5 cm ± 0.6 % (0,50) | 8.4 cm ± 0.3 % (0,23) | 8.2 cm ± 0.6 % (-0,04) | 7.3 cm ± 0.3 % (0,52) |
|  | Standard ASiR-V 80 (N&A) | 12.2 cm ± 0.4 % (0,49) | 10.9 cm ± 0.4 % (0,33) | 8.8 cm ± 0.4 % (0,19) | 8.7 cm ± 0.6 % (0,23) | 8.5 cm ± 0.6 % (0,61) | 8.4 cm ± 0.3 % (0,23) | 8.2 cm ± 0.6 % (0,02) | 7.3 cm ± 0.3 % (0,66) |
|  | Standard TF low (N&A) | 12.2 cm ± 0.4 % (0,49) | 10.9 cm ± 0.4 % (0,31) | 8.8 cm ± 0.4 % (0,51) | 8.7 cm ± 0.6 % (0,36) | 8.4 cm ± 0.6 % (0,62) | 8.4 cm ± 0.4 % (0,42) | 8.2 cm ± 0.7 % (0,02) | 7.3 cm ± 0.4 % (0,53) |
|  | Standard TF medium (N&A) | 12.2 cm ± 0.4 % (0,46) | 10.9 cm ± 0.4 % (0,20) | 8.8 cm ± 0.3 % (0,33) | 8.7 cm ± 0.7 % (0,35) | 8.4 cm ± 0.6 % (0,47) | 8.4 cm ± 0.3 % (0,30) | 8.2 cm ± 0.8 % (0,20) | 7.3 cm ± 0.4 % (0,57) |
|  | Standard TF high (N&A) | 12.2 cm ± 0.4 % (0,50) | 10.9 cm ± 0.4 % (0,32) | 8.8 cm ± 0.3 % (0,47) | 8.7 cm ± 0.7 % (0,17) | 8.4 cm ± 0.6 % (0,48) | 8.4 cm ± 0.4 % (0,19) | 8.2 cm ± 0.8 % (0,05) | 7.3 cm ± 0.4 % (0,52) |
|  |  |  |  |  |  |  |  |  |  |
| Scanner 2 | QR40 Safire 0 (N&A) | 12.2 cm ± 0.6 % (0,38) | 10.8 cm ± 0.6 % (0,39) | 8.6 cm ± 0.6 % (0,54) | 8.6 cm ± 0.8 % (0,31) | 8.4 cm ± 0.9 % (0,46) | 8.4 cm ± 0.6 % (0,26) | 8.3 cm ± 1.1 % (0,20) | 7.5 cm ± 0.7 % (0,39) |
|  | QR40 Safire 3 (N&A) | 12.2 cm ± 0.6 % (0,30) | 10.8 cm ± 0.6 % (0,49) | 8.7 cm ± 0.7 % (0,25) | 8.7 cm ± 0.7 % (0,20) | 8.4 cm ± 0.8 % (0,50) | 8.4 cm ± 0.6 % (0,24) | 8.3 cm ± 1.0 % (0,35) | 7.5 cm ± 0.7 % (0,49) |
|  | QR40 Safire 5 (N&A) | 12.2 cm ± 0.6 % (0,37) | 10.8 cm ± 0.6 % (0,57) | 8.6 cm ± 0.6 % (0,48) | 8.7 cm ± 0.6 % (0,37) | 8.4 cm ± 0.9 % (0,25) | 8.4 cm ± 0.5 % (0,05) | 8.3 cm ± 0.8 % (0,37) | 7.5 cm ± 0.6 % (0,32) |
|  | QR40 Safire 0 (L-S) | 12.1 cm ± 1.1 % (0,33) | 10.6 cm ± 0.9 % (0,48) | 8.4 cm ± 0.8 % (0,38) | 8.4 cm ± 1.5 % (0,33) | 8.2 cm ± 1.4 % (0,48) | 8.1 cm ± 0.8 % (0,29) | 8.1 cm ± 1.7 % (0,35) | 7.1 cm ± 0.9 % (0,41) |
|  | QR40 Safire 3 (L-S) | 12.1 cm ± 0.9 % (0,29) | 10.6 cm ± 0.7 % (0,48) | 8.3 cm ± 0.8 % (0,35) | 8.4 cm ± 1.2 % (0,42) | 8.1 cm ± 1.2 % (0,49) | 8.1 cm ± 0.8 % (0,20) | 8.0 cm ± 1.4 % (0,40) | 7.1 cm ± 0.8 % (0,33) |
|  | QR40 Safire 5 (L-S) | 12.1 cm ± 0.9 % (0,50) | 10.6 cm ± 0.7 % (0,52) | 8.3 cm ± 0.8 % (0,38) | 8.4 cm ± 1.2 % (0,24) | 8.1 cm ± 1.3 % (0,58) | 8.1 cm ± 0.7 % (0,19) | 8.0 cm ± 1.5 % (0,17) | 7.1 cm ± 0.8 % (0,20) |
|  | QR40 Safire 0 (DirectSPR) | 12.3 cm ± 0.8 % (0,36) | 10.8 cm ± 0.7 % (0,62) | 8.6 cm ± 0.7 % (0,39) | 8.7 cm ± 1.0 % (0,39) | 8.4 cm ± 1.3 % (0,44) | 8.4 cm ± 0.7 % (0,21) | 8.3 cm ± 1.2 % (0,40) | 7.5 cm ± 0.8 % (0,56) |
|  | QR40 Safire 3 (DirectSPR) | 12.4 cm ± 0.8 % (0,35) | 10.8 cm ± 0.7 % (0,08) | 8.7 cm ± 0.8 % (0,16) | 8.7 cm ± 0.9 % (0,28) | 8.5 cm ± 1.3 % (0,43) | 8.5 cm ± 0.8 % (0,14) | 8.4 cm ± 1.2 % (0,38) | 7.6 cm ± 0.8 % (0,26) |
|  | QR40 Safire 5 (DirectSPR) | 12.3 cm ± 0.7 % (0,36) | 10.8 cm ± 0.6 % (0,37) | 8.6 cm ± 0.7 % (0,25) | 8.7 cm ± 0.9 % (0,24) | 8.4 cm ± 1.2 % (0,39) | 8.4 cm ± 0.6 % (0,24) | 8.4 cm ± 1.1 % (0,30) | 7.5 cm ± 0.6 % (0,20) |

Table S18. Mean range with 87% confidence interval (± 1.5 SD) and skewness (within parenthesis) for protons passing through tissue equivalent phantom inserts in simulations based on SPR maps generated from image data acquired with 15 mGy CTDI_vol._ SPR maps were generated with N&A [13] or L-S [15,16], with different levels of iterative noise reduction (ASiR-V / Safire) or deep-learning based noise reduction (TF). Abbreviations: SD = standard deviation; SPR = stopping power ratios; N&A = Näsmark & Andersson (2021); L-S = Landry-Saito; CTDI_vol_ = computed tomography dose index; TF = True Fidelity.

|  | **Kernel and noise reduction Lung (inhale)** | | **Lung (exhale)** | **Adipose** | **Breast** | **Liver** | **Muscle** | **Bone 200** | **Bone 800** |
| --- | --- | --- | --- | --- | --- | --- | --- | --- | --- |
| Scanner 1 | Standard ASiR-V 0 (N&A) | 12.2 cm ± 0.4 % (0,52) | 10.9 cm ± 0.4 % (0,54) | 8.8 cm ± 0.4 % (0,39) | 8.7 cm ± 0.5 % (0,37) | 8.4 cm ± 0.6 % (0,32) | 8.4 cm ± 0.3 % (0,29) | 8.2 cm ± 0.7 % (0,17) | 7.3 cm ± 0.4 % (0,32) |
|  | Standard ASiR-V 40 (N&A) | 12.2 cm ± 0.4 % (0,47) | 10.9 cm ± 0.3 % (0,63) | 8.8 cm ± 0.3 % (0,27) | 8.7 cm ± 0.5 % (0,22) | 8.4 cm ± 0.6 % (0,51) | 8.4 cm ± 0.4 % (0,34) | 8.2 cm ± 0.6 % (0,17) | 7.3 cm ± 0.4 % (0,37) |
|  | Standard ASiR-V 80 (N&A) | 12.2 cm ± 0.4 % (0,50) | 10.9 cm ± 0.4 % (0,56) | 8.8 cm ± 0.4 % (0,36) | 8.7 cm ± 0.5 % (0,35) | 8.4 cm ± 0.6 % (0,27) | 8.4 cm ± 0.4 % (0,17) | 8.2 cm ± 0.8 % (0,00) | 7.3 cm ± 0.3 % (0,48) |
|  | Standard TF low (N&A) | 12.2 cm ± 0.4 % (0,63) | 10.9 cm ± 0.3 % (0,53) | 8.8 cm ± 0.3 % (0,33) | 8.7 cm ± 0.6 % (0,22) | 8.4 cm ± 0.5 % (0,43) | 8.4 cm ± 0.5 % (0,35) | 8.2 cm ± 0.7 % (0,13) | 7.3 cm ± 0.5 % (0,40) |
|  | Standard TF medium (N&A) | 12.2 cm ± 0.4 % (0,68) | 10.9 cm ± 0.4 % (0,58) | 8.8 cm ± 0.4 % (0,24) | 8.7 cm ± 0.6 % (0,33) | 8.4 cm ± 0.5 % (0,29) | 8.4 cm ± 0.5 % (0,08) | 8.2 cm ± 0.7 % (0,09) | 7.3 cm ± 0.4 % (0,50) |
|  | Standard TF high (N&A) | 12.2 cm ± 0.4 % (0,56) | 10.9 cm ± 0.3 % (0,54) | 8.8 cm ± 0.4 % (0,27) | 8.7 cm ± 0.6 % (0,30) | 8.4 cm ± 0.5 % (0,31) | 8.4 cm ± 0.5 % (0,22) | 8.2 cm ± 0.6 % (0,13) | 7.3 cm ± 0.5 % (0,55) |
|  |  |  |  |  |  |  |  |  |  |
| Scanner 2 | QR40 Safire 0 (N&A) | 12.2 cm ± 0.6 % (0,46) | 10.7 cm ± 0.6 % (0,52) | 8.6 cm ± 0.5 % (0,44) | 8.6 cm ± 0.7 % (0,13) | 8.4 cm ± 1.0 % (0,60) | 8.4 cm ± 0.5 % (0,03) | 8.3 cm ± 1.1 % (0,22) | 7.5 cm ± 0.7 % (0,31) |
|  | QR40 Safire 3 (N&A) | 12.2 cm ± 0.6 % (0,35) | 10.8 cm ± 0.6 % (0,58) | 8.7 cm ± 0.5 % (0,57) | 8.7 cm ± 0.6 % (0,24) | 8.4 cm ± 1.0 % (0,68) | 8.4 cm ± 0.5 % (0,14) | 8.3 cm ± 1.0 % (0,15) | 7.5 cm ± 0.6 % (0,34) |
|  | QR40 Safire 5 (N&A) | 12.2 cm ± 0.6 % (0,39) | 10.8 cm ± 0.6 % (0,65) | 8.7 cm ± 0.5 % (0,26) | 8.7 cm ± 0.7 % (0,28) | 8.4 cm ± 0.9 % (0,50) | 8.4 cm ± 0.5 % (-0,18) | 8.3 cm ± 1.0 % (0,17) | 7.5 cm ± 0.6 % (0,23) |
|  | QR40 Safire 0 (L-S) | 12.1 cm ± 1.1 % (0,42) | 10.5 cm ± 0.8 % (0,86) | 8.4 cm ± 0.8 % (0,67) | 8.4 cm ± 1.1 % (0,33) | 8.1 cm ± 1.6 % (0,75) | 8.1 cm ± 0.8 % (0,11) | 8.0 cm ± 1.7 % (0,19) | 7.1 cm ± 0.8 % (0,36) |
|  | QR40 Safire 3 (L-S) | 12.1 cm ± 0.9 % (0,23) | 10.5 cm ± 0.7 % (0,87) | 8.4 cm ± 0.6 % (0,45) | 8.4 cm ± 1.3 % (0,56) | 8.1 cm ± 1.5 % (0,87) | 8.1 cm ± 0.7 % (0,23) | 8.0 cm ± 1.6 % (0,42) | 7.1 cm ± 0.7 % (0,43) |
|  | QR40 Safire 5 (L-S) | 12.1 cm ± 0.8 % (0,27) | 10.6 cm ± 0.7 % (0,52) | 8.4 cm ± 0.6 % (0,40) | 8.4 cm ± 1.4 % (0,37) | 8.1 cm ± 1.3 % (0,51) | 8.1 cm ± 0.6 % (0,38) | 8.1 cm ± 1.8 % (0,23) | 7.1 cm ± 0.8 % (0,45) |
